# Structural and Functional Insights Into Skl and Pal Endolysins, Two Cysteine-Amidases With Anti-pneumococcal Activity. Dithiothreitol (DTT) Effect on Lytic Activity

**DOI:** 10.3389/fmicb.2021.740914

**Published:** 2021-10-29

**Authors:** Cristina Gallego-Páramo, Noelia Hernández-Ortiz, Rubén M. Buey, Palma Rico-Lastres, Guadalupe García, J. Fernando Díaz, Pedro García, Margarita Menéndez

**Affiliations:** ^1^Instituto de Química-Física Rocasolano, Consejo Superior de Investigaciones Científicas, Madrid, Spain; ^2^Centro de Investigación Biomédica en Red de Enfermedades Respiratorias (CIBERES), Instituto de Salud Carlos III (ISCIII), Madrid, Spain; ^3^Metabolic Engineering Group, Universidad de Salamanca, Salamanca, Spain; ^4^Centro de Investigaciones Biológicas Margarita Salas, Consejo Superior de Investigaciones Científicas, Madrid, Spain

**Keywords:** endolysin, anti-pneumococcal activity, CHAP domain, amidase_5 domain, choline-binding domain, cysteine-peptidase, reducing agents, DTT-mediated activation

## Abstract

We have structurally and functionally characterized Skl and Pal endolysins, the latter being the first endolysin shown to kill effectively *Streptococcus pneumoniae*, a leading cause of deathly diseases. We have proved that Skl and Pal are cysteine-amidases whose catalytic domains, from CHAP and Amidase_5 families, respectively, share an α_3_β_6_-fold with papain-like topology. Catalytic triads are identified (for the first time in Amidase_5 family), and residues relevant for substrate binding and catalysis inferred from *in silico* models, including a calcium-binding site accounting for Skl dependence on this cation for activity. Both endolysins contain a choline-binding domain (CBD) with a β-solenoid fold (homology modeled) and six conserved choline-binding loci whose saturation induced dimerization. Remarkably, Pal and Skl dimers display a common overall architecture, preserved in choline-bound dimers of pneumococcal lysins with other catalytic domains and bond specificities, as disclosed using small angle X-ray scattering (SAXS). Additionally, Skl is proved to be an efficient anti-pneumococcal agent that kills multi-resistant strains and clinical emergent-serotype isolates. Interestingly, Skl and Pal time-courses of pneumococcal lysis were sigmoidal, which might denote a limited access of both endolysins to target bonds at first stages of lysis. Furthermore, their DTT-mediated activation, of relevance for other cysteine-peptidases, cannot be solely ascribed to reversal of catalytic-cysteine oxidation.

## Introduction

Endolysins are encoded by bacteriophages (phages) as part of their lytic system to allow the phage progeny to exit the host bacterial cell. These enzymes lyse bacterial cells by cleaving the covalent bonds connecting the building blocks of the peptidoglycan (PG) network, which results in bacterial lysis. Exogenous addition of recombinant endolysins results in rapid lysis and death of susceptible bacteria (lysis from without). Endolysins have thus emerged as a novel class of antibacterials of use in human and animal health, food preservation, and agriculture protection (Love et al., 2018; São-José, 2018) that have now entered clinical trial stages (Gerstmans et al., 2018). Their distinctive features as antibacterials include: (*i*) selective and rapid killing of specific bacteria leaving the surrounding commensal microbiota virtually intact; (*ii*) activity in numerous environments with independence of bacterial growth phase; (*iii*) unlikely development of resistances; (*iv*) synergism with other lysins and standard antibiotics; and (*v*) easily engineered to create novel enzymes with tailored profiles (activity, specificity, stability and solubility) (Loeffler et al., 2001; Gerstmans et al., 2018; São-José, 2018). All these unique properties make endolysins the ideal candidates to eradicate multidrug-resistant pathogens and prevent further resistance developments.

Modularity is a key feature for endolysins selectivity and customization. Lysins from Gram-positive-infecting bacteriophages often comprise at least one catalytic domain with amidase, endo/peptidase, glycosidase, or lytic transglycosylase activity, and one or more binding domains which recognize specific epitopes present in the cell wall (Vázquez et al., 2021; and references herein). Catalytic domains targeting the same chemical bond of PG can belong to different families, and a broad diversity of cell wall binding domains and endolysin architectures have been identified as well (Bustamante et al., 2017; Gerstmans et al., 2018; Vázquez et al., 2021). Thus, together with the specific structure of the PG and the bacterial envelope, the combination of domains and their arrangement within endolysin overall structure determines the site of cleavage and the range of susceptible bacteria (Díez-Martínez et al., 2013, 2015; Regulski et al., 2013; São-José, 2018). Endolysins from Gram-negative phages are frequently single domain proteins, but multidomain proteins have been reported (Vázquez et al., 2021).

**FIGURE 1:**
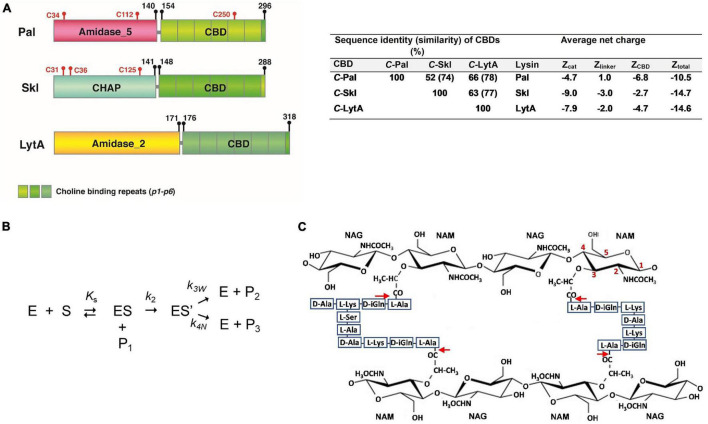
**(A)** Schematic representation of Pal, Skl, and LytA NAM-amidase structures. Domains [*Amidase_5* (PF05382), *CHAP* (PF05257), *Amidase_2* (PF01510)], choline-binding repeats (PF01473), and linkers are specified by different colors. Initial and final amino acids and cysteine positions are indicated. Sequence similarity between domains and average net charges are summarized on the right side. **(B)** Schematic representation of the reaction pathway for the hydrolysis by cysteine-peptidases. E and S are the free enzyme and the substrate, ES the non-covalent enzyme-substrate complex, ES’ the acyl-enzyme formed by the nucleophilic attack of the catalytic cysteine, P_1_ the leaving amine product, P_2_ the acyl product formed by water attack, and *K*_*s*_, *k*_2_, and *k*_3w_ the dissociation constant of the non-covalent complex, the acylation constant, and the water-mediated deacylation constant, respectively. P_3_ is the product resulting from the attack of the acyl-enzyme by other nucleophile, and *k*_4w_ the respective first-order rate constant. **(C)** Schematic representation of pneumococcal peptidoglycan. Red arrows indicate the cleavage sites for NAM-amidases. NAG and NAM stand for *N*-acetylglucosamine and *N*-acetylmuramic acid. Cross-linking of stem peptides takes place, mainly, by Ser–Ala, and Ala–Ala dipeptides, or by direct binding of D-Ala to L-Lys moieties. Teichoic acids are covalently attached to position 6 of NAM.

*Streptococcus pneumoniae* is a human Gram-positive pathogen included in the WHO priority group of multidrug resistant bacteria (World Health Organization [WHO], 2017). Overall, this bacterium causes around 1.2 million deaths per year, most of which occur in developing countries (GBD 2017 Causes of Death Collaborators, 2018). Pal, the *N*-acetylmuramoyl-L-alanine amidase (NAM-amidase) encoded by pneumococcal Dp-1 bacteriophage, was the first endolysin reported to efficiently kill pneumococci of every serotype tested, including penicillin-resistant isolates (Loeffler et al., 2001; Jado et al., 2003). It comprises an *N*-terminal catalytic domain (N-Pal) of the *Amidase_5* family, and a C-terminal CBD (C-Pal) (Figure 1) targeting the (lipo)teichoic acids of the pneumococcal cell wall, which determines the range of susceptible bacteria. LytA, the major pneumococcal autolysin, and Skl, the endolysin of the *Streptococcus mitis* prophage φSK137, are representative of the other two types of NAM-amidases able to digest the pneumococcal cell wall (Llull et al., 2006). Their catalytic domains, of the *Amidase_2* and cysteine/histidine-dependent amidohydrolase/peptidase (CHAP) families, respectively, are fused to a C-Pal homologous CBD made of six sequence-conserved repeats (*p1*–*p6*) and a C-terminal tail (Figure 1). According to the crystal structure of LytA (Fernández-Tornero et al., 2001; Li et al., 2015), the CBD would fold into a left-handed β-solenoid, where the repeats and the C-terminal tail form a β-hairpin each. Choline binds at the interface of two consecutive repeats and promotes LytA and Pal dimerization, which in LytA takes place by pairing of the two last β-hairpins from each CBD (Fernández-Tornero et al., 2001; Li et al., 2015) and is determinant for the activity (Varea et al., 2000). Interestingly, the most lethal NAM-amidase so far tested against *S. pneumoniae* is PL3, a chimera of Pal and LytA in which the last four repeats and the C-tail of Pal were substituted by those of LytA (Blázquez et al., 2016).

Successful elimination of *S. pneumoniae* by Pal and LytA has been thoroughly investigated (Loeffler et al., 2001; Jado et al., 2003; Rodríguez-Cerrato et al., 2007a,b; Domenech et al., 2011), as well as choline-binding affinity and self-association equilibria (Varea et al., 2000; Varea et al., 2004; Rodríguez-Cerrato et al., 2007a). However, neither the antipneumococcal potential of Skl nor its interaction with choline has been evaluated. In addition, little is known about Pal and Skl three-dimensional (3D) structure, substrate recognition, and catalysis. Although the atomic structure of several CHAP domains has been solved (McGowan et al., 2012; Gu et al., 2014; Sanz-Gaitero et al., 2014; Zhou et al., 2020), the 3D structure of an *Amidase_5* domain is yet unknown, though a distant relationship with the NlpC/P60 family (Pfam entry PF00877) has been proposed (Anantharaman and Aravind, 2003). CHAP and NlpC/P60 domains belong to the Peptidase_CA clan (CL0125), whose members are evolutionarily related to the papain-like cysteine-protease family (Anantharaman and Aravind, 2003; Bateman and Rawlings, 2003; Ridgen et al., 2003). All of them adopt an α+β-fold, contain conserved cysteine and histidine residues, and most likely operate by a nucleophilic attack mechanism with the conserved cysteine as the catalytic nucleophile (Figure 1; Storer and Ménard, 1994). Both CHAP and Amidase_5 domains are well represented within endolysin sequences available in databases (Vázquez et al., 2021).

Using a combination of computational, mutational, biophysical, and biochemical approaches, we have characterized Skl–choline interactions, choline-mediated self-association, and its pneumococcal bactericidal activity. In parallel, *in silico* models of Pal and Skl domains have been generated and their structural features explored with relation to substrate recognition by the catalytic cavity, choline-binding capacity, and dimer formation. 3D models of the overall structure of Pal and Skl in solution are also proposed from SAXS studies. Additionally, the origin of the sigmoidal profiles of bacteriolysis and the effect of DTT on lytic activity, of relevance for other endolysins and for cysteine-dependent endolysins, respectively, have been explored and the results discussed in mechanistic terms.

## Materials and Methods

### Bacterial Strains

All bacterial strains used in this study (Supplementary Table 1) were stored at −80°C. *Escherichia coli* strains were grown in LB medium at required temperatures with shaking. Ampicillin (100 μg/ml) was added if needed. *S. pneumoniae* was grown at 37°C without shaking in C medium supplemented with 0.08% (w/v) yeast extract (C+Y medium), except strain 48 that was incubated in brain heart infusion (BHI) broth (Becton, Dickinson, and Company) supplemented with albumin 0.008% (v/v).

### Constructs, Protein Expression, and Purification

Plasmids used in this study are listed in Supplementary Table 2. Pal-coding region was PCR amplified using pMSP11 as template (Sheehan et al., 1997) and Pal5-Fw and DL2-Rv as primers. The *skl* gene was obtained by digestion of pFSK6 (Llull et al., 2006) with *Xba*I and *Hind*III restriction enzymes. Then, *pal* and *skl* genes were subcloned into pT7-7 plasmid previously digested with *Xba*I and *Hind*III, and the resulting plasmids (pSkl and pPal) were used as templates to construct Skl C36S and C125S mutants, and Pal C34A, H99A, H111A, C112S, and C250S mutants. Skl C36S–C125S and Pal C112S–C250S double mutants were constructed using pSkl-C36S and pPal-C250S as templates, respectively. Site-directed mutagenesis was performed with Pfu DNA polymerase (Biotools) (Wang and Wilkinson, 2000) using appropriate oligonucleotides (Supplementary Table 2). Synthetic DNA fragments coding for Skl catalytic mutants (C31A, H92A, and E109A) were purchased from ATG:biosynthetics (Merzhausen, Germany) as pUC-derivative recombinant plasmids that were subcloned into pT7-7, using *Nde*I and *Pst*I restriction sites. pT7-7 derivatives were maintained in *E. coli* DH5α or DH10B cells before transformation into *E. coli* BL21(DE3) or C41(DE3) cells for protein overproduction. Skl Y264H was a spontaneous mutant isolated during retransformation of pFSK6 into BL21 (DE3) cells. Mutations were confirmed by DNA sequencing (SecuGen S.L.) of recombinant plasmids. Expression and purification of Skl and Pal variants were performed as reported for the wild-type (WT) forms (Llull et al., 2006; Varea et al., 2004), with slight variations. Briefly, after induction with IPTG and overnight incubation using appropriate conditions (Supplementary Table 2), cells were harvested by centrifugation (5,250× *g*, 30 min), suspended in 20 mM phosphate buffer (PB), pH 7, and lysed using a French press. Cell debris was removed by centrifugation (27,000× *g*, 45 min), and the cleared lysate was supplemented with NaCl (1 M final concentration) and then loaded onto a DEAE-cellulose column equilibrated in PB, pH 7.0. The purified protein was eluted with 0.28 M choline chloride, 0.1 M NaCl PB, after exhaustive washing of the matrix with 1.5 M NaCl PB (0.1 M NaCl PB for Skl E109A mutant). All purification steps were performed at 4°C and the purity and masses of isolated proteins were checked by SDS-PAGE and MALDI-TOF (Bustamante et al., 2017) before storage at −20°C. Proteins were dialyzed at 4°C against the appropriate buffer and centrifuged for 5 min at 11,600× *g* before use, unless otherwise stated. Protein concentration was determined spectrophotometrically using the theoretical molar absorption coefficient at 280 nm (ProtParam; http://web.expasy.org/protparam/; 1 μM equals to 0.0335 mg/ml Skl and 0.0345 mg/ml Pal). Choline concentration was measured by differential refractometry (Medrano et al., 1996).

### Activity Measurements on Pneumococcal Cell Walls

Assays for cell-wall lytic activity were carried out according to standard conditions (Díez-Martínez et al., 2013) using [*methyl*-^3^H]choline-labeled pneumococcal cell walls as substrate in PB or PB supplemented with 10 mM DTT (PB_*DTT10*_). Results are the mean of two to seven independent experiments, usually made in triplicate.

### Bacteriolytic and Bactericidal Assays

Bacteria grown to logarithmic phase (OD_550_ of 0.3) were washed with the reaction buffer, and the final OD_550_ adjusted to ca. 0.6 in the same buffer containing (or not) DTT. Activity was assayed in 96-well plates (Falcon) by adding the enzyme (25 μl) to the bacterial suspension (225 μl). Samples were incubated at 37°C with a brief shaking every 5 min, and the OD_550_ decrease following enzyme addition was measured in a VERSAmax Microplate Reader (Molecular Devices, San Jose, CA, United States). Controls were always run in parallel substituting the added enzyme by buffer. Sigmoidal profiles of bacteriolysis were fitted to the Boltzmann equation (**OD_550_**(**t**) = {**A_2_** + (**A_1_**−**A_2_**)/[**1** + **exp**((**t**−**t_0_**)/**dt**)]}) using the Origin 6.0 software, after correcting for the OD_550_ decrease of controls. A_1_ and A_2_ are OD_550_ values when t → 0 and ∞, respectively; t_0_ is the center of the sigmoid; and the time constant dt determines the sigmoid maximal slope, which was used to calculate the maximal lytic activity [(**A_2_**−**A_1_**)/**4dt**] and lag duration (**t_0_**−**2dt**). After incubation, bacterial viability (CFU/ml) was determined in blood agar plates, and colonies were counted after overnight incubation at 37°C, as described (Blázquez et al., 2016). Typically, three separate determinations were carried out, each of them in triplicate. Statistical analysis was performed using GraphPad InStat v. 6.0 (GraphPad Software, San Diego, CA, United States) and is detailed in figure captions. In all cases, *p* < 0.05 values were considered statistically significant.

### Circular Dichroism

Circular dichroism (CD) measurements were performed in PB buffer at 20°C in a Peltier-controlled J-810 spectropolarimeter (Jasco Corp., Tokyo, Japan) using protein concentrations of 3–5 μM (far-UV) and 10–15 μM (near-UV). Spectra were recorded and analyzed as described (Varea et al., 2004). Choline-titration experiments were carried out collecting spectra at increasing ligand concentration, upon serial addition of small volumes of choline-concentrated stocks to the same protein sample (Blázquez et al., 2016). The ellipticity change at the selected wavelength was plotted as a function of choline concentration, and the dissociation constants were calculated by non-lineal least square fitting of titration curves assuming two independent sets of binding sites, each of them filled according to the Hill equation:


$$\theta =\theta_{0}+\sum_{i=1,2}\left(\theta_{i}-\theta_{i-1}\right)\times \left[1/\left(1+{\left({\text{K}}_{0.5\,\mathrm{app}}^{i}/\left[L\right]\right)}^{a_{i}}\right)\right]$$

where θ_0_ and θ are the ellipticity values in the absence and presence of a choline concentration [*L*], respectively, (θ_*i*_ − θ_*i*__–__1_) the ellipticity change induced by saturation of sites *i*, and *K*^*i*^_0.5_*_*app*_* and *a*_*i*_ the apparent half-dissociation constant and the binding cooperativity of these sites.

### Analytical Ultracentrifugation

Ultracentrifugation experiments were carried out at 20°C in an Optima XL-A analytical ultracentrifuge (Beckman Coulter, Brea, CA, United States) as previously described (Díez-Martínez et al., 2015). Equilibrium sedimentation experiments were performed at different rotor speeds, and mass conservation in the cell was checked in all the experiments, that were analyzed with the Heteroanalysis program^1^ to the sedimentation model for a single species. Differential sedimentation coefficients, C(*s*), were calculated by least-squares boundary modeling of sedimentation velocity profiles, and normalized to values in water (*s*_20,w_) with the SEDFIT program (Schuck, 2000). The Stokes radii (*R*_*s*_) and the frictional coefficient ratio (*f*/*f*_0_), related to the protein size and hydrodynamic shape, were calculated with the SEDNTERP program (Laue et al., 1992), as reported elsewhere (Díez-Martínez et al., 2015).

### Secondary-Structure Prediction

Prediction of secondary structure was performed using Prof^2^, PSIPRED^3^, and JPred4^4^ servers. Final predictions only considered residues whose secondary structure was forecasted to be more than 86% reliable by at least two methods.

### Molecular Modeling of Skl and Pal Modules

Identification of structural homologs of Skl and Pal modules was carried by scanning the query sequence against the PDB with DELTA-BLAST (Altschul et al., 1997) using an *E*-value cutoff of 10^–4^. Only matches with most significant *E*-values were retained. The structures with the greatest identity and sequence coverage showing the best correlation with the target predicted secondary structure were selected as templates. Sequence alignments were performed with Clustal_X and MODELLER 9.13 (Thompson et al., 1997; Eswar et al., 2006), followed by manual editing guided by template structure and query secondary-structure prediction, and functional amino acids. 3D models of each module containing all non-hydrogen atoms were constructed with MODELLER using as input such alignments. Initially, 20 models of each module were automatically generated, but only those with the lowest energy and fewest restrains violations were selected for further optimization using ModRefiner (Xu and Zhang, 2011^5^) and 3DRefine (Bhattacharya et al., 2016^6^). When sequence identity between the query and the potential templates identified with DELTA-BLAST was <40%, threading methods [Phyre2 (Kelley et al., 2015), HHPred (Söding et al., 2005), and I-TASSER (Roy et al., 2010)] were used in parallel to detect distant homologs, and the best models (built with MODELLER in the case of HHpred- or Phyre2-identified templates) were also minimized, when pertinent. Final models were structurally and energetically evaluated with PROCHECK (Laskowski et al., 1993) and VERIFY 3D (Lüthy et al., 1992) using the Verification Server^7^. The geometry of catalytically relevant residues and choline-binding amino acids in models and templates was also compared. Choline molecules were transferred to C-Pal and C-Skl final models from the crystal structure of C-LytA (PDB entry 4IWT) and CbpF (site 2; PDB entry 2X8O). Sequence conservation studies in N-Pal and N-Skl were carried out with the ConSurf server (Berezin et al., 2004^8^) that use CSI-BLAST to retrieve sequences from the Uni-ref90 database (150 sequences) and align sequences with MAFFTL-INS-I (minimal and maximal sequences identities were 35 and 95%, respectively). Structure figures were made using Pymol 1.3^9^.

### Small-Angle X-ray Scattering

Small-angle X-ray scattering (SAXS) measurements were performed at 4°C at the BM16 station of ESRF (Grenoble, France). Before data acquisition, the proteins were exhaustively dialyzed against the appropriate buffer and centrifuged to remove possible aggregates (Bustamante et al., 2017). The camera covered ranges from 0.01 to 0.25 Å^–1^ of the scattering vector, *q*, defined as 4πsin(θ)/λ, where θ is the scattering angle. Absolute *q* values were obtained by reference to the orders of the 670 Å repeat in wet rat tail collagen. Protein spectra were acquired at three different concentrations (2–8 mg/ml) in the absence and presence of choline. No changes by radiation damage were detected in the 30 frames of 30 s each recorded per sample, which were averaged and normalized by beam intensity and detector response before subtracting buffer scattering from protein data using ATSAS package (Petoukhov et al., 2012). Corrected spectra were then normalized and extrapolated to infinite dilution with ATSAS, used also for data processing and analysis. The radius of gyration (*R*_*g*_), the pair-of distances-distribution function [*P*(*r*)], the maximum intraparticle distance (*D*_*max*_), and the Porod’s volume were determined as described (Bustamante et al., 2017). Next, 10 *ab initio* bead models of each protein were constructed with DAMMIN/DAMMIF (Svergun, 1999; Franke and Svergun, 2009) with and without P2 symmetry restrictions for protein dimers. The models were superimposed with SUPCOMB (Petoukhov et al., 2012), filtered by spatial discrepancy with DAMFILT (spatial discrepancy parameter was 0.57 ± 0.07 Å), and averaged with DAMAVER (Volkov and Svergun, 2003). Rigid body modeling of Pal and Skl structures was performed with BUNCH and SASREF programs (Petoukhov and Svergun, 2005) using as input the coordinates of the 3D models built, separately, for the catalytic domain and the CBD. The theoretical scattering profiles of SAXS-derived models were calculated with CRYSOL (Svergun et al., 1995). BUNCH and SASREF models were automatically adjusted to the low resolution DAMMIM/F envelopes with SUPCOMB.

### PAGE-SDS Analysis

Proteins at 0.36 mg/ml were pretreated in the dark, at room temperature, with 40 mM iodoacetamide for 30 min, and then incubated with 1% (v/v) SDS for other 30 min. Finally, sample buffer with or without β-mercaptoethanol (SB_β_ and SB, respectively) was added to the samples before being loaded in 12% polyacrylamide gels (1.5 μg protein per well). Controls without iodoacetamide pretreatment were run in parallel, and gels were stained with Coomassie blue.

## Results and Discussion

### Skl-choline Interaction: Ligand-Induced Structural Changes

**FIGURE 2:**
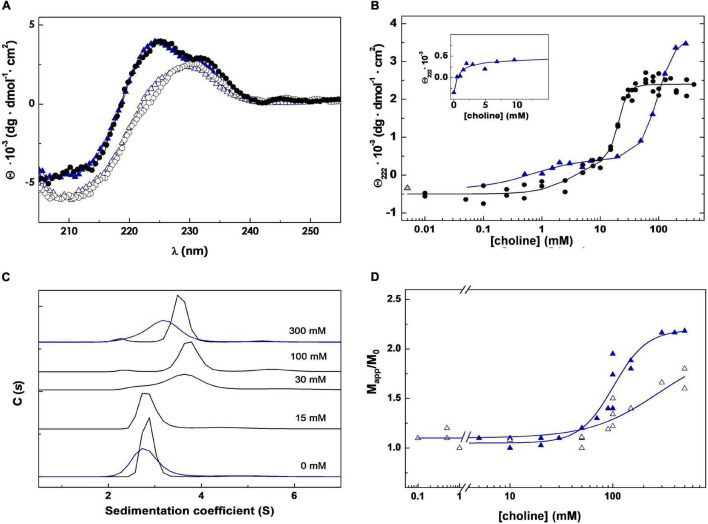
Choline binding to Skl NAM-amidase. **(A)** Far-UV CD spectra of Skl WT (black circles) and the Y264H mutant (blue triangles) in the absence and presence of 300 mM choline (open and solid symbols, respectively). **(B)** CD choline titration into Skl WT (circles) and Y264H mutant (triangles) monitored by measuring the ellipticity variation at 222 nm as a function of ligand concentration. Solid lines are the theoretical fit of experimental data with the Hill equation extended to two sets of sites. The first phase of the Y264H titration curve is shown in the inset. **(C)** Monomer-dimer distribution of Skl WT (black lines) and Y264H mutant (blue lines) at choline concentrations indicated in panel labels, determined by sedimentation velocity under conditions of titration experiments. C(s) represents the sedimentation coefficient distribution. **(D)** Dependence of the relative molecular mass (Mapp/M0) of Skl Y264H mutant with choline concentration at pH 6.5 and 8.0 (open and full symbols, respectively). Mapp is the average molecular mass determined by equilibrium sedimentation and M_0_ the monomer molecular mass. Data were acquired at 20°C in PB (pH 6.5), except for measurements at pH 8.0 **(D)**, performed in 50 mM Tris–HCl buffer.

The titration of Skl with choline was monitored by CD following the ellipticity increase promoted by ligand binding in the region of 220–230 nm (Figure 2A), sensitive to the chiral contribution of the aromatic side chains conforming choline-binding sites. The titration curve, obtained by plotting the ellipticity increase at 222 nm as a function of choline concentration, shows a biphasic profile consistent with the presence of two sets of binding sites (Figure 2B). The sharp ellipticity rise observed between 10 and 30 mM choline also evidenced that saturation of the lower affinity sites was highly cooperative. The analysis of the binding curve using the Hill equation to fit the saturation of each set of sites (see ***CD method section***) yielded apparent half-dissociation constants, *K*_0.5,*app*_, of 4 and 20 mM, and Hill coefficients, *a*_*i*_, of 1 and 5.5, for the higher and lower affinity sites, respectively. Analytical ultracentrifugation was employed next to determine the oligomerization state of Skl, its dependence on choline concentration, and the hydrodynamic parameters of the protein species in solution. The sedimentation coefficient distribution showed a major species (>90%) compatible with a monomer (*s*_20,*w*_ = 2.87 ± 0.02 S; M = 33.3 kDa) in the absence of choline and also upon saturation of the higher affinity sites. As ligand binding to the lower affinity sites proceeded, a peak compatible with a dimer (*s*_20,w_ = 3.85 ± 0.03 S; M = 62.3 kDa) was detected and became the main species after site saturation (Figure 2C). A minor species (≤5%) with a *s*_20,w_ of 4.7 S, which shifted up to 5.9 ± 0.3 S at 30 mM choline and above, was also noted. The molecular masses of unbound Skl (monomer) and its choline-saturated complex (dimer) were confirmed by sedimentation equilibrium experiments (33.6 ± 0.2 kDa and 65.7 ± 3 kDa, respectively).

The value of *f*/*f*_0_ of both species, calculated from sedimentation velocity data, differed significantly from each other (1.33 vs. 1.57) and from the value expected for globular particles. A great increase of *R*_*s*_ (Stokes radius) from 28.5 to 44.0 Å occurred also upon dimerization. Collectively, these data evidenced that both species have elongated structures, and that Skl dimerization does not occur through lateral association of the monomers. Of note, the hydrodynamic parameters of the Skl dimer in complex with choline are similar to those of Pal (*s*_20,w_ = 3.9 S; *f*/*f*_0_ = 1.60; *R*_*S*_ = 44.2 Å) and LytA (*s*_20,w_ = 4.1 S; *f*/*f*_0_ = 1.56; *R*_*S*_ = 43.8 Å) dimers (Blázquez et al., 2016).

### Three-Dimensional Modeling of Pal and Skl Domains

#### Modeling of Pal Catalytic Domain (N-Pal)

**FIGURE 3:**
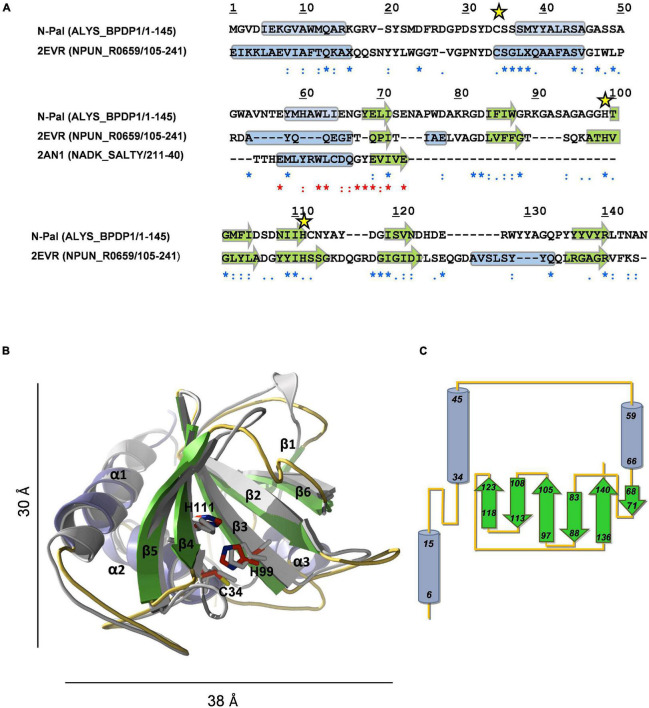
Structural model of N-Pal. **(A)** Sequence alignment of the target and the templates (PDB entries: 2EVR and 2AN1) used to build N-Pal 3D model with MODELLER. UniProtKB accession numbers and regions comprised in the alignment are shown in parenthesis. Asterisks, colons, and dots stand for strictly conserved residues, conservative, and semiconservative substitutions, respectively, with relation to 2EVR (blue) or 2AN1 (red) sequences. Yellow stars indicate the catalytic residues. Secondary structural elements of template structures, and sequence-based predicted for N-Pal are indicated by cylinders (helices) and arrows (β-strands). **(B)** Structural superimposition of the N-Pal model generated with MODELLER (cartoon representation; helices in light blue, arrows in green, and loops in orange) and the 2EVR template (gray). Catalytic residues, in stick representation, are colored in gray (template) or by element (N-Skl; C: red, N: blue; S: yellow). For the sake of clarity, the helix of 2EVR previous to β6 is not shown. **(C)** Topology diagram of N-Pal model.

The search of structural homologues confirmed a clear, albeit distant, relationship of N-Pal with the catalytic domains of bacterial enzymes belonging to the N1pC/P60 family. The N1pC/P60 domain from the putative γ-D-glutamyl-L-diamino acid-endopeptidase of *Nostoc punctiforme* PCC 73102 (NPUN_R0659; PDB entry 2EVR) (Xu et al., 2009) showed the longer sequence coverage and the highest sequence similarity and secondary-structure correlation with the target. However, the region encompassing predicted helix-3 and β strand-1 of N-Pal aligned poorly with that of 2EVR, and search of databases identified residues 21–40 of a putative kinase from *Salmonella typhimurium* LT2 (PDB entry 2AN1) as the sequence-closest structurally known fragment. Accordingly, both fragments were used simultaneously as templates to model N-Pal with MODELLER. The Ramachandran plot of the best final model−obtained with the alignment shown in Figure 3A−showed 99.2% residues in allowed regions, 0.8% in disallowed regions, and the energetic evaluation with VERIFY 3D compared well with that of the 2EVR template (97.3 and 99.5% residues had 3D-1D averaged scores ≥ 0.2, respectively). According to the model, N-Pal comprises three α-helices and a six-stranded antiparallel β-sheet in an α_3_β_6_ topology with strand β6 located between strands β1 and β2 (Figures 3B,C). Given the low sequence identity (23.4%) of the target and the templates, several threading methods were used in parallel to model N-Pal but only the model provided by I-TASSER compared in quality (99.2% residues in allowed regions, and 93.2% residues with 3D-1D scores ≥ 0.2) with that from MODELLER. Of note, the closest related structures identified by I-TASSER belonged all to the NlpC/P60 family. Main differences between both *in silico* models were located in: (*i*) helix-3, partially rotated and much longer than sequence-predicted in the I-TASSER model; and (*ii*) the segment-connecting strands β5 and β6, predicted as a loop by MODELLER and sequence-based methods but built as a 5-residue long helix by ITASSER (Supplementary Figures 1A,B). The highly conserved Cys-His-His catalytic triad of the NlpC/P60 family was structurally conserved in both models (Figure 3; Supplementary Figure 1C), with Cys34 located at the beginning of helix-2 and packed against the *β-*sheet core, near to His99 from β3 strand and His111 from *β4* strand. However, the relative orientation of the triad side chains in the model from MODELLER is closer to the disposition found in the 2EVR template and related structures (Supplementary Figure 1C). Implication of Cys34, His99, and His111 residues in catalysis was confirmed by mutational analysis. Individual substitution by alanine produced correctly folded mutants, as confirmed by CD and sedimentation velocity (Supplementary Figure 2), and practically abrogated the hydrolytic activity of the C34A mutant, reducing to 4 and 36% those of H99A and H111A mutants, respectively (Supplementary Table 3). Notably, a ConSurf analysis of sequence conservation across the *Amidase_5* family showed that the highly conserved residues of N-Pal are clustered in a shallow groove in the center of which is located the catalytic triad (Supplementary Figure 3A). The channel runs along one side of the catalytic domain and is asymmetrically lined with neutral polar and charged residues from strands β2, β3, and β4, and the loops connecting strands β2-β3, β4-β5, β5-β6, and helices α1-α2 and α2-α3 (Supplementary Figure 3B) that conform the substrate-binding surface according to the model. The network of catalytic residues comprises the side chains of Cys34 and His99, likely acting as a thiolate-imidazolium ion-pair catalytic nucleophile, and His111, located at H-bond distance of His99 that will help to properly orient the side chain of His99 (Supplementary Figure 3C). The distance of 3.6 Å between the sulfur atom of Cys34 and the Nδ1 atom of His99 in the N-Pal model, somewhat high for a hydrogen bond, compares well with the average value of 3.7 ± 0.2 Å found in the NlpC/p60 structures accessible in the PDB. On the other hand, the phenol group of highly conserved residue Tyr21, close to the catalytic triad, could participate in substrate binding and, then, in stabilization of the developing anionic tetrahedral intermediate during catalysis, as the equivalent Tyr41 moiety does in the complex of the NlpC/P60-related amidase domain of AmiA of *Bacteroides uniformis* with GlcNAc-anhMurNAc, by hydrogen bonding the lactic group of the ligand (Xu et al., 2014; PDB entry 4Q5K).

#### Modeling of Skl Catalytic Domain (N-Skl)

**FIGURE 4:**
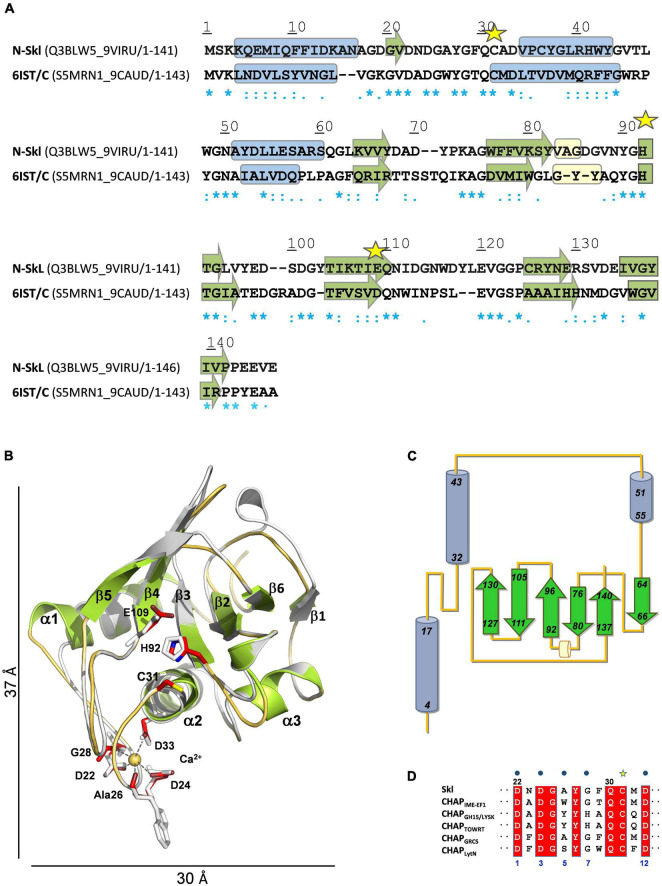
Structural model of N-Skl. **(A)** Sequence alignment of the target and the template (PDB entry: 6IST) used by MODELLER. UniProtKB accession numbers and regions comprised in the alignment are shown in parenthesis. Symbols, cylinders, and arrows are as in Figure 3A. **(B)** Structural superimposition of the N-Skl model generated with MODELLER (cartoon representation: helices and arrows in green, loops in orange, and Ca^2+^ cation yellow sphere) and the template (in gray). Catalytic and Ca^2+^-coordinating residues shown in stick representation are colored by element (C: red, N: blue; S: yellow) in N-Skl, or gray in the template. **(C)** Topology diagram of *N*-Skl structural model. **(D)** Sequence alignment of the 12-residue Ca^2+^-binding loop; blue dots indicate the Ca^2+^-coordinating residues (positions 1, 3, 5, 7, and 12).

The search of structural homologues of N-Skl identified the CHAP domain of the *Enterococcus faecalis* phage IME-EF1 endolysin (CHAP_*IME–EF1*_; PDB entry 6IST) (Zhou et al., 2020) as the best possible template (full sequence coverage; 31% sequence identity and 62% similarity). The Ramachandran plot of the final model−obtained with the alignment of Figure 4A−showed that all residues were in allowed regions (88.4% in most favored regions) and according to VERIFY 3D 87% residues had a 3D-1D average score ≥ 0.2 (88.8% in 6IST template). N-Skl model displays the α_3_β_6_ topology predicted above for N-Pal plus a short 3_10_-helix between strands β2 and β3 (Figures 4B,C). The similarity of fold displayed by both catalytic domains would be consistent with the presence of a positive band centered ∼230 nm in the far-UV CD spectra of both NAM-amidases (Figure 2A; Supplementary Figure 2A), which does not appear in the spectra of choline-binding proteins (CBPs) with another type of catalytic domain (Varea et al., 2000; Díez-Martínez et al., 2015). The putative catalytic triad (Cys31, His92 and Glu109) is located in a broad and long groove (Figure 4B; Supplementary Figure 4A), at positions similar to those of the Pal triad, and surrounded by highly conserved residues, as shown in the analysis of molecular conservation using the ConSurf server (Supplementary Figure 4B). Implication in catalysis was confirmed by mutational analysis. C31A and H92A substitutions produced well-folded proteins, as confirmed by CD and analytical ultracentrifugation (Supplementary Figures 5A–C), and reduced the lytic activity to 6 and 1.4% of Skl WT, respectively (Supplementary Table 3). Unexpectedly, E109A substitution resulted in an impaired retention of the mutant in DEAE columns, time-dependent loss of the CD native-like spectra in solution, and propensity to oligomerization (Supplementary Figures 5D,E), unveiling a key role of this residue in Skl structural stability and folding. In our model, Glu109 is at the center of a complex network of feasible hydrogen bonds (Supplementary Figure 4C), whose disruption might account for all these facts. Besides, the carboxylic oxygen of Glu109 could help to orientate His92 side chain (at hydrogen-bond distance) and partially withdrew the positive charge of the imidazolium cation, during the attack of the amide bond by the thiolate-imidazolium ion-pair formed with Cys31. The distance of 3.4 Å between the Nδ1 atom of His92 and the sulfur atom of Cys31 compares well with values (3.8 ± 0.3 Å on average) found in most structures of CHAP domains available in the PDB, as also does His92 orientation. Interestingly, the equivalent imidazolium ring has a reversed orientation in some crystallographic forms of the CHAP domain of the *E. coli* glutathionylspermidine synthase/amidase (GspS) and in the CHAP domain of the PlyC endolysin (Pai et al., 2011; McGowan et al., 2012), pointing to its capability to be placed in varying optimal positions along the different steps of the catalytic process, as it happens during papain catalysis (Storer and Ménard, 1994).

The bottom of the catalytic groove, formed by residues from helix α2, strands β3 and β5, and the loop connecting α2 to α3 (Supplementary Figures 4C,D), is flanked by the long loops connecting α1 to α2 and strands β4 to β5, on one side, and α2 to α3 and β2 to β3, on the other, which can help direct the entry of the substrate and confer specificity to the binding site. Additionally, the side chains of Gln30 and Asn111, and the backbone NH groups of Gly49 and Gly91−all highly conserved in CHAP domains and located near the catalytic triad−could participate through hydrogen bonding in substrate binding and, then, in stabilization of the tetrahedral anionic intermediate, as the equivalent amino acids (Gln58, Gly79, Gly130, and Asn149) do in the CHAP amidase domain of *E. coli* GSP (Pai et al., 2011).

Similarly, to CHAP_*IME–EF1*_ and other CHAP domains, Skl requires Ca^2+^ cations for activity (Llull et al., 2006). According to our model, eight out of the 12 amino acids conforming the Ca^2+^-binding site of CHAP_*IME–EF1*_ would be sequence and structurally conserved in Skl (Figures 4B,D), including four of the five calcium-coordinating residues (positions 1, 3, 7, and 12). Besides, Ca^2+^ coordination by the fifth residue (position 5) takes place through the oxygen atom of the main chain and its change to alanine would likely preserve calcium binding (Gu et al., 2014; Sanz-Gaitero et al., 2014). Thus, Ca^2+^ coordination by Asp22, Asp24, Ala26, Gly28, and Asp33 could help maintain the structure of the long loop connecting helices α1 and α2, and so modulate the conformation of the catalytic groove. Finally, the side chains of Tyr27 (conserved in Ca^2+^-dependent CHAP domains; Figure 4D) and Tyr90 might also contribute to direct the entrance and the recognition of the substrate (Supplementary Figure 4D). Remarkably, a tyrosine is present at the position of Tyr90 in more than 90% of the CHAP domains encoded by bacteria from the *Firmicutes* phylum (Rossi et al., 2008).

#### Homology Modeling of Pal and Skl CBDs (C-Skl and C-Pal)

**FIGURE 5:**
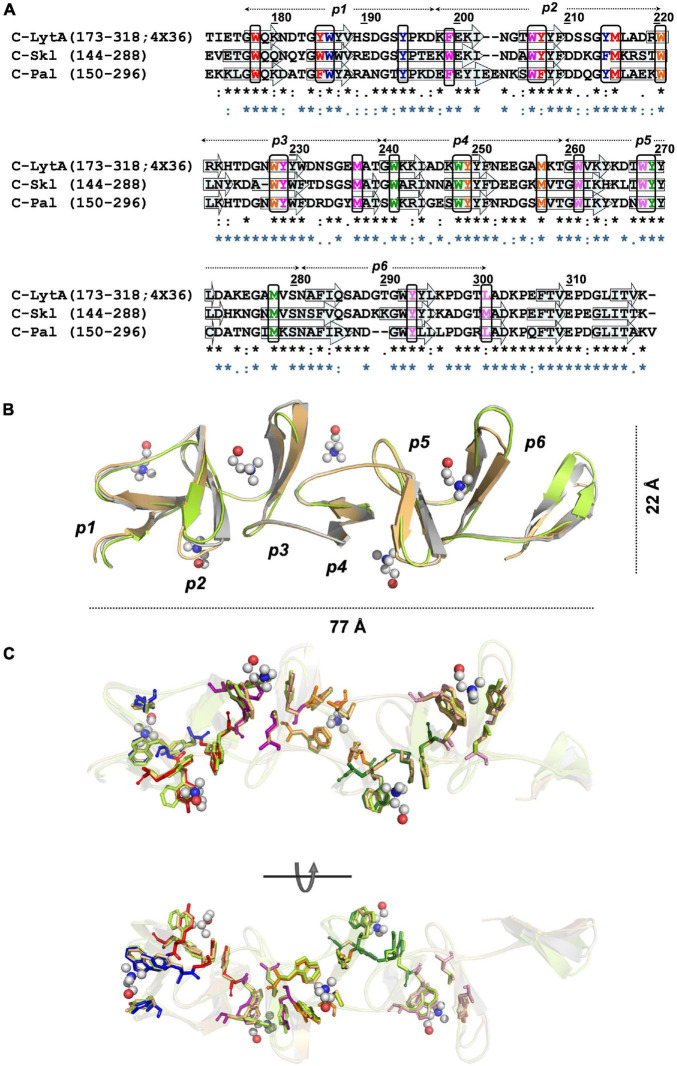
Structural models of Skl and Pal CBDs. **(A)** Sequence alignment of C-Skl and C-Pal targets with 4X36 C-LytA template. Arrows indicate the positions of β-strands in C-LytA structure, and sequence-based predicted for targets. Black rectangles enclose residues involved in choline binding colored by site (1: blue, 2: red, 3: magenta, 4: orange, 5: green, 6: pink). Symbols at the bottom show sequence conservation of C-Skl (black) and C-Pal (blue) in relation to the template. **(B)** Structural superposition of final 3D models and the 4X36 template, in cartoon representation, colored in light green (C-Skl), light orange (C-Pal), and gray (C-LytA). **(C)** Structural conservation of residues involved in choline binding depicted in stick representation [color code: C-Skl (light green), C-Pal (light orange), and C-LytA (by site, as in **(A)**). Choline molecules are depicted in the ball-and-stick model (C: gray, O: red, N: blue).

The structure of the CBD of LytA from the pneumococcal TIGR4 strain (Mellroth et al., 2014; PDB entry 4X36) and the pneumococcal prophage SPH_0121 (Li et al., 2015; PDB entry 4IWT) were identified as the closest homologs of C-Skl and C-Pal. Template sequences differ from each other in eight amino acids (four of them conservative changes) and provide full sequence coverage of the target (63–66% sequence identities; Figure 5A). The 3D models built with MODELLER using them separately as templates were virtually identical, and Figure 5 illustrates the final models generated with 4X36 as template. Conformational evaluation showed that 99.2% residues of C-Pal and C-Skl models were in allowed regions (only one residue of the first β-hairpin turn was in disallowed regions), and all residues had an average score ≥ 0.2 in the energetic evaluation with VERIFY 3D. The models consist of seven β-hairpins−corresponding to the six repeats (*p*1–*p*6) and the C-terminal tail−that fold into the left-handed β-solenoid characteristic of C-LytA and other closely related structures (PDB entries: 1H09, 2BIB, 2V04, and 2WW5). As shown in Table 1 and Figure 5, the type of residues defining the five feasible canonical loci (sites 2–6) and the non-canonical site 1 are sequentially and structurally conserved in both models. Also, the hydrophobic binding pockets recognizing the choline-methyl groups are correctly formed between each two consecutive repeats, and no steric clashes were detected upon choline transfer to respective loci. These findings, altogether, indicate that Pal and Skl could bind also up to six moieties of choline per monomer, while CD titration experiments showed that the three NAM-amidases exhibit two different sets of binding sites (Medrano et al., 1996; Varea et al., 2004). However, full saturation of Pal (Varea et al., 2004) and Skl required a concentration of choline 1.5- and 3.0-times higher than LytA (Medrano et al., 1996), respectively, and dimerization was coupled to saturation of the higher affinity sites in LytA and of the lower affinity sites in Pal or Skl. Such differences could denote: (*i*) slight variations in choline-mediated specific contacts, including the electrostatic interactions between the positive charge of the ligand and the differentially charged surfaces of the CBDs (Figure 1) and/or (*ii*) disparities in the propensity of CBDs for choline-induced dimerization.

**TABLE 1:** Residues conforming choline-binding sites of Skl and Pal.

Site	Repeats	Skl				Pal			
1^a^	*p*1–*p*2	W157	Y165	F185	–	W163	Y171	Y193	–
2	*p*1–*p*2	W149	W156	Y178	M186	W155	F162	F186	M194
3	*p*2–*p*3	W170	W177	Y199	M207	F176	W185	Y208	M215
4	*p*3–*p*4	W191	W198	Y219	M227	W199	W207	Y228	M235
5	*p*4–*p*5	W211	W218	Y239	M248	W220	Y227	Y248	M257
6	*p*5–*p*6	W231	W238	Y263	M271	W240	W247	Y270	L278

*^*a*^Non-canonical site found in the CBD of LytA (Mellroth et al., 2014; Li et al., 2015) and previously predicted in Cpl-1 (Buey et al., 2007).*

### Dimerization Interface of Skl and Pal

LytA dimerization involves, primarily, the creation of a network of hydrophobic contacts between the C-tail and the *p6* β-hairpins of the two monomers (Fernández-Tornero et al., 2001, 2002; Li et al., 2015). Two residues, Tyr294 and Ile315, are especially relevant for the hydrophobic interactions, which embrace three zones (Fernández-Tornero et al., 2002). As shown in Table 2, all residues relevant for C-LytA dimerization are conserved in C-Skl, except Val317 (Thr287 in Skl). Like Skl, the V317T mutant of LytA is monomeric in the absence of choline and its full dimerization requires alike choline concentrations (Romero et al., 2007). These facts, along with the large increment of *f*/*f*_0_ and *R*_*S*_ derived from Skl dimerization (see above), strongly points to a tail-to-tail LytA-like dimer (Supplementary Figure 6). To further substantiate this proposal, we examined the effect of substituting Tyr264 (equivalent to key Tyr294 residue of LytA) by histidine on choline-binding affinity and choline-induced dimerization, assessing first that the Skl WT structure was preserved in the mutant using CD (Figure 2A). As expected, introduction of a charged imidazolium ring drastically altered Skl Y264H dimerization (∼50% of the mutant remained as a monomer under choline saturating conditions at pH 6.5) and the apparent affinity of the lower affinity sites was significantly reduced as well, exposing two well-defined plateaus on the titration curve (Figure 2B). Moreover, full dimerization of the Y264H mutant was reached at pH 8.0 where the neutral form of histidine predominates and unfavorable electrostatic interactions are eliminated (Figure 2D). Collectively, current results are consistent with the implication of the C-terminus of Skl in dimerization. Still, the required choline concentration was 10-fold higher than in Skl WT, unveiling a differential effect of tyrosine and neutral histidine side chains in the stabilization of the hydrophobic core.

**TABLE 2:** Residues of *p*_6_ and the C-tail relevant for dimer formation.

Hydrophobic contacts											
**Zone 1**				**Zone 2**					**Zone 3**		
LytA^a^	F283	W292	**Y294**	P305	F307	V309	**I315**	V317	S286	Y293	L314
Skl	F253	W262	**Y264**	P275	F277	V279	**I285**	T287	S256	Y263	L284
Pal	F262	W269	**L271**	P282	F284	V286	**I292**	A294	Y265	Y270	L292
**Intermolecular hydrogen bonds**											
LytA^a^	W292-I315 (double)			Y293-D312							
Skl	W262-I285 (double)			Y263-E282							
Pal	W269-I292 (double)			Y270-D289							

*^*a*^From Fernández-Tornero et al. (2002). Residues especially relevant for C-LytA dimerization are in bold and those not conserved in C-Pal or C-Skl are underlined. In blue, residues from the other monomer of the dimer.*

Choline-induced dimerization of Pal would likely occur through tail-to-tail interactions of the CBD as well, considering the high conservation of relevant residues in C-Pal (Table 2), and the great similarity of the *f*/*f*_0_ and *R*_*S*_ values of Pal, LytA, and Skl dimers. As in Skl, substitution of alanine for valine at position 294 of Pal could alter the contacts within the hydrophobic core (zone 2), as might also happen with the change Tyr→Leu at position 271 (zone 1). In addition, the turn connecting the two strands of the *p6* repeat of Pal is two amino acids shorter than in C-LytA and C-Skl, due to the absence of the G289T290 pair (LytA numbering), which, together with the change Ser→Tyr at position 265 (beginning of the turn; zone 3), might somewhat alter the spatial orientation of *p*6 and the C-tail β-hairpin in the dimer of Pal, and/or the dimerization angle. On the other hand, the possibility of compensating sequence changes in the last two β-hairpins of Pal cannot be discarded, since Pal full dimerization takes place at about two times lower concentration of choline than Skl.

### SAXS-based Overall 3D Structure of Pal and Skl

**FIGURE 6:**
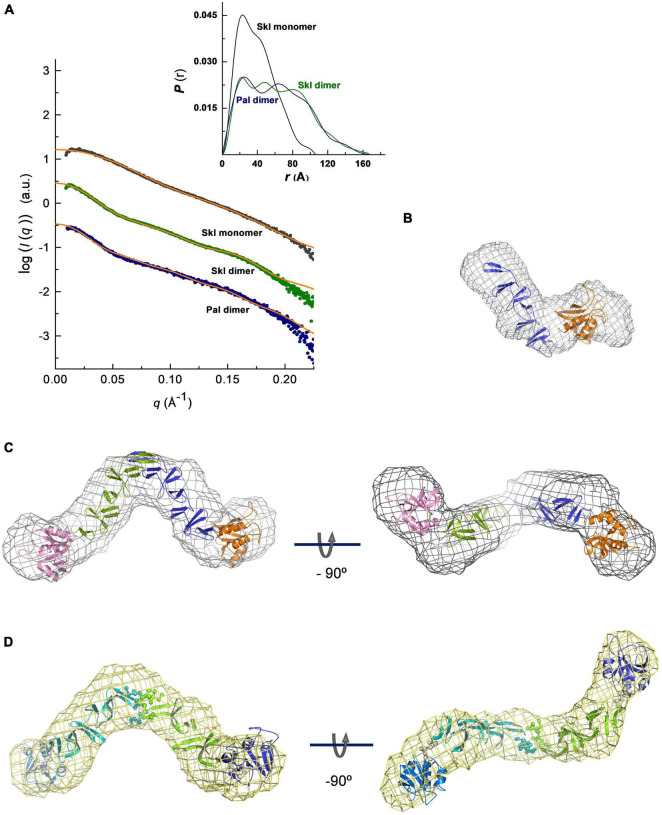
SAXS-based overall structures of Pal and Skl NAM-amidases. **(A)** Overlay of the scattering curves at infinite dilution of Skl and Pal (symbols) with the fits obtained by rigid-body modeling (orange traces). χ^2^ values between experimental and theoretical data calculated with CRYSOL were 0.646 (Skl), 0.512 (Skl/choline), and 0.153 (Pal/choline). The inset shows the real-space distance-distribution functions *P*(r), calculated with GNOM, for the respective configurations in solution. **(B–D)** *Ab initio* models built with DAMMIF/DAMMIN (mesh representation) superimposed with best rigid models (cartoon representation) of the Skl monomer **(B)**, dimer **(C),** and Pal dimer **(D)**. Views of rigid-body models of Pal and Skl dimers unveil the *trans* configurations of the NAM-amidase domains located at the extremes of the molecule (N-Skl: orange and pink domains; N-Pal: blue and violet domains; spheres depict dummy residues assigned to avoid clashes at the dimer interface and linker amino acids). Measurements were made at 4°C in 20 mM PB (Pal) or 50 mM Tris–HCl (Skl) at pH 8.0, supplemented or not with choline.

To investigate domain disposition within the monomer structure and further characterize the dimerization mode, the solution overall structure of Pal and Skl was characterized by SAXS. The scattering curves were registered at three different protein concentrations in the absence (Skl) or in the presence of saturating concentrations of choline (Skl and Pal). The coexistence of different oligomerization states prevented to characterize Pal by SAXS in the absence of choline. The scattering profiles extrapolated at infinite dilution and the pair-of-distance distribution functions *P*(*r*) are depicted in Figure 6A. The molecular masses estimated from Porod volumes agreed with the molecular species identified by analytical ultracentrifugation. The unbound form of Skl, with a radius of gyration (*R*_*g*_) ∼31 Å and a maximal intraparticle dimension (*D*_*max*_) of 105 Å, shows a bimodal profile of *P*(*r*) with two distinctive maxima at *r* values of 23 and 40 Å (Figure 6A, inset), reflecting the modular structure of the Skl monomer. Dimer formation results in a large increase of the particle dimensions (*R*_*g*_ = 50 Å; *D*_*max*_ = 168 Å) and a third peak appeared in the *P*(*r*) curve at ∼80 Å. The scattering curve and the *P*(*r)* function of the Pal dimer in complex with choline, with a *R*_*g*_ value of 49 Å and a *D*_*max*_ of 160 Å, closely resemble that of the Skl dimer. Collectively, these data reflect: (*i*) the elongated shape of the monomer and dimer species, (*ii*) the large increment of the particle length upon dimerization, and (*iii*) the structural similarity of Pal and Skl dimers, and they are consistent with ultracentrifugation results and the dimerization mode proposed in ***Dimerization interface of Skl and Pal***. Indeed, the *ab initio* models of Pal and Skl in complex with choline reconstructed from SAXS data showed a V-shaped scaffold with two globular domains attached to the ends (Figures 6C,D) that closely resembled the structures of LytA and Cpl-1 muramidase dimers (Buey et al., 2007; Mellroth et al., 2014; Li et al., 2015), despite the fact that Cpl-1 dimerization occurs through head-to-head interactions of the CBDs. This way of dimerization was consequence of the domain disposition within the Cpl-1 monomer, which was determined by: (*i*) a long, extended linker that houses a negatively charged DDEEDD motif at the N-terminus, genuine of Cpl-1, and (*ii*) the unusual disposition of the last three β hairpins of the CBD in a six-stranded β-sheet that inserts in a hydrophobic cavity of the catalytic module, shielding the C-terminal end of the CBD from the solvent (Hermoso et al., 2003; Li et al., 2015). Moreover, the low-resolution model generated for the Skl monomer in the absence of choline (Figure 6B) correlated well with the size and shape of each monomer within the dimer model, where the elongated part of each monomer would correspond to the CBD and the globular part to the catalytic domain. Rigid-body models were also constructed using the 3D models built for the isolated domains (Figures 6B–D). The best dimeric models (lowest divergence of SAXS experimental profile and the theoretical curve calculated from the model; χ-value) were obtained using the two catalytic domains and the CBD dimer as individual rigid bodies and assuming P2 symmetry. The atomic models of C-Skl and C-Pal dimers were based on the crystal structures of LytA and C-LytA dimers in the PDB entries 4X36 and 1H8G, respectively. Additionally, the amino acids of the C*-*tail of C-Pal were introduced as dummy residues for calculations, in order to avoid steric clashes at the interface of the dimer (not clashes occurred at the interface of Skl dimer, depicted in Supplementary Figure 6). Rigid-body models agreed well with the *ab initio* models (Figure 6A) and revealed that the amidase domains of Pal and Skl dimers were positioned in *trans* configuration with respect to the plane defined by the two subunits of the CBD; the internal angle between them being somewhat lower in the Skl dimer (∼90°) than in Pal dimer (∼110°).

### Biochemical Characterization of Skl Antipneumococcal Activity

**FIGURE 7:**
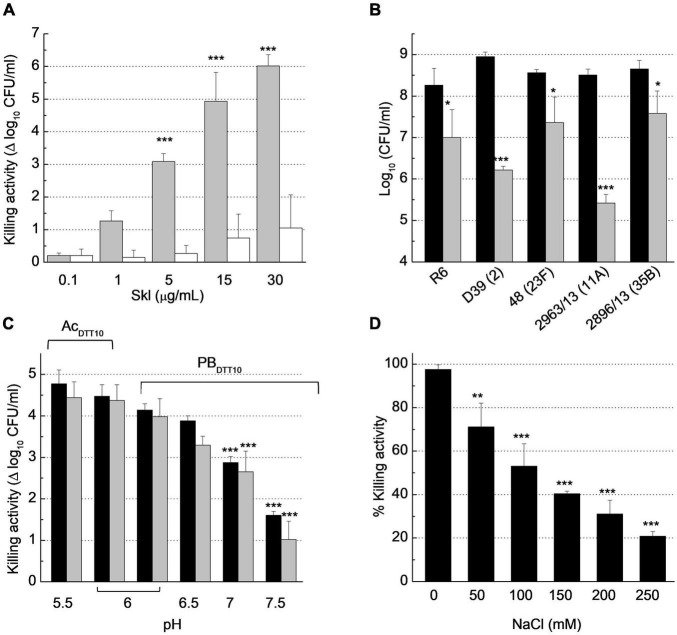
Biochemical characterization of Skl anti-pneumococcal activity. Bacterial suspensions were treated with buffer (control) or with Skl for 60 min before dilution and plating on blood agar for viable cell determination (CFU/ml). **(A)** Dose-dependent decrease of *S. pneumoniae* R6 viability upon treatment with Skl WT or the Y264H mutant (gray and white bars, respectively) in PBS_*DTT10*_ (137 mM NaCl, 2.7 mM KCl, 10 mM Na_2_HPO_4_, 1.8 mM KH_2_PO_4_, 10 mM DTT, pH 6.8) at 37°C. **(B)** Killing activity of Skl WT (1 μg/ml) against various pneumococcal strains (PBS_*DTT10*_, 37°C) (serotypes indicated in parenthesis; black bars represent controls). **(C)** Effect of pH on the killing activity of Skl (2.5 μg/ml) against D39 strain, measured at 30°C (black bars) and 37°C (gray bars) in PB_*DTT10*_ or Ac_*DTT10*_ buffer (20 mM sodium acetate, 10 mM DTT). **(D)** Influence of NaCl concentration on the bactericidal activity of Skl (1 μg/ml) against D39 cells (PB_*DTT10*_, pH 6.0, 30°C). Statistical analysis for **(A,C)** was performed using two-way ANOVA followed by Sidak post-test to compare Skl WT and the Y264H mutant, and with PB_*DTT10*_ (pH 6.0) at the same temperature, respectively. No significant differences were found between 30 and 37°C. In **(B)**, treated and untreated bacteria were compared by a two-tailed *t*-test, and in **(D)** one-way ANOVA was performed followed by Dunnet’s post-test taking 0 mM NaCl as reference. Error bars represent standard deviations from three independent experiments. Asterisks mean a significant difference (*p < 0.05; **p < 0.01; ***p < 0.001).

The antibacterial capacity of Skl was tested against several pneumococcal strains using the protocol described in ***Bacteriolytic and bactericidal assays***, which measures the OD_550_ decrease of bacterial suspensions as a function of time, and bacterial survival at the end of incubation in the absence (control) and presence of endolysin. Skl was active, in a concentration- and strain-dependent manner, on the five pneumococcal strains tested (Figures 7A,B), which included the non-encapsulated R6 strain and the parental encapsulated strain D39 (serotype 2), clinical isolates representatives of two emergent non-PCV13 serotypes with high potential for biofilm formation (2963/13, serotype 11A; and 2896/13, serotype 35B), and the multiresistant 48 strain (serotype 23F). After 60 min, a single dose of 1 μg/ml of Skl decreased the viable titer of D39 and 2963/13 strains by 3 logs, and those of strains R6, 48, and 2896/13 by ∼1.2 logs, as compared with controls incubated with buffer alone (PBS_*DTT10*_, pH 6.8, 37°C). The killing capacity of Skl WT increased by about two additional logs at moderate acidic pH (Figure 7C) and was favored also by a low salt concentration (Figure 7D). Besides, the lysis proceeded somewhat faster at 30°C but no statistically significant differences were found in the killing capacity after 60 min incubation in a given buffer (Figure 7C). As a whole, our results showed that optimal antipneumococcal activity of Skl compares well with those of Pal and LytA under equivalent conditions, and with those of ClyJ-3 and ClyJ-3m, two variants of a chimera built by fusing the CHAP domain of PlyC and the choline-specific CBD of the gp20 endolysin from the *Streptococcus* phage SPSL1 (Luo et al., 2020; Yang et al., 2020). Interestingly, the Y264H substitution reduced the bactericidal activity of Skl by several orders of magnitude (Figure 7A), further supporting the relevance of dimerization for the activity. Curiously, the antipneumococcal activity of ClyJ-3 was improved by deletion of the C-terminal tail of the CBD in the ClyJ-3m monomeric variant, which might denote a less favorable interaction of the highly active PlyC domain with the cleavable peptidoglycan bonds upon dimerization of this fully engineered lysin (Luo et al., 2020).

Remarkably, the time course profiles of Skl were sigmoidal when the reaction conditions reduced the velocity of lysis (e.g., low protein concentration, NaCl addition, higher temperature), being appropriately described by the Boltzmann equation (see “Materials and Methods”). The initial slow exponential phase in the OD_550_ decrease determines the duration of the lag phase, which is followed by an approximately linear stage of maximal velocity and a final phase where the curve asymptotically approaches the final OD_550_ value (Supplementary Figure 7). At high lysis velocity, the initial phase is probably shortened, making it imperceptible.

### Insight on the Bacteriolytic Action of Skl and Pal: Influence of DTT

#### Evaluation of Disulfide Bridge Formation

**FIGURE 8:**
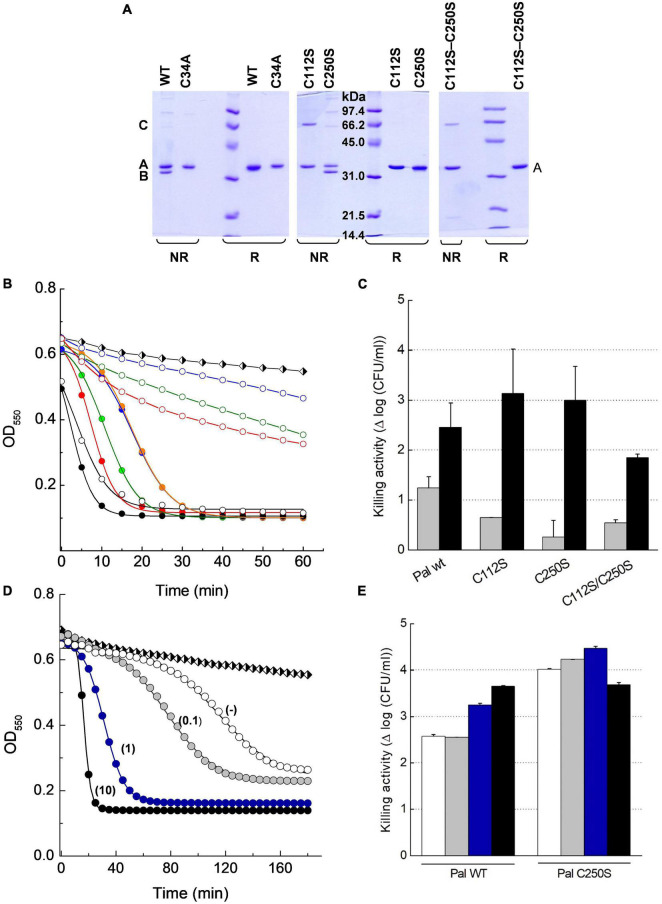
Insight on DTT effect on Pal lytic activity. **(A)** SDS-PAGE analysis of Pal WT and its Cys mutants under reducing (R) and non-reducing (NR) conditions. Samples pretreated with iodoacetamide were incubated in sample buffer with (SB_β_) and without (SB) β-mercaptoethanol for 3 min before the analysis. Bands B and A correspond to the Pal monomer with and without an intramolecular Cys34–Cys112 disulfide bridge, respectively, and band C to a dimer with a Cys34–Cys34 intermolecular disulfide. **(B)** Lytic activity of Pal variants (5 μg/ml) against pneumococcal R6 strain in PB and PB_*DTT10*_, pH 6.8, 37°C (open and solid symbols, respectively). Turbidity decay was monitored as a function of time for 60 min. Symbols and solid lines depict representative experimental data after correction for control decay and the fits to the Boltzmann sigmoid, respectively (black: WT; green: C112S; blue: C250S; red: C112S–C250S; C250S preincubated in PB_*DTT10*_: orange). Black and white diamonds in **(B,D)** illustrate control decay. **(C)** Killing activity of Pal variants in PB and PB_*DTT10*_ (gray and black bars, respectively) in samples from **(B)** after 60 min incubation. Error bars represent standard deviations from two to three independent experiments each of them in triplicate. **(D)** Effect of DTT concentration (figure labels; mM units) in the lysis buffer on the lag period and the maximum velocity of R6 lysis by Pal C250S (5 μg/ml) preincubated in PB_*DTT10*_. Symbols and solid lines depict representative data, and they mean as in **(B)**. **(E)** Killing activity of Pal WT and C250S mutant in samples from **(D)** after 3 h incubation (white, gray, blue, and black bars stand for no DTT (−), 0.1, 1, and 10 mM DTT, respectively). Error bars represent standard deviations from duplicates of a single experiment.

**FIGURE 9:**
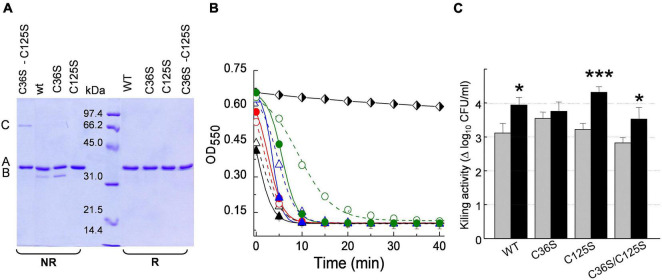
Insight on DTT effect on Skl catalytic activity. **(A)** SDS-PAGE analysis of Skl WT and its Cys mutants under reducing (R) and non-reducing (NR) conditions (samples treated as in Figure 8A). Bands B and A correspond to the monomer with and without an intramolecular Cys31–Cys125 disulfide bridge, and band C to a dimer with a Cys31–Cys31 intermolecular disulfide. **(B)** Lytic activity of Skl variants (5 μg/ml) against R6 strain in PB and PB_*DTT10*_ at pH 6.8 and 37°C (open and solid symbols, respectively). Symbols and solid lines depict representative experimental data after correction for control decay and fit to the Boltzmann sigmoid, respectively (WT: black triangles; C36S: red circles; C112S: blue triangles; C112S–C250S: green circles). Black and white diamonds illustrate control decay. **(C)** Killing activity of Skl variants in PB and PB_*DTT10*_ (gray and black bars, respectively) in samples from **(B)** after 60 min incubation. Error bars represent standard deviations from three independent experiments. Asterisks mean a significant difference (*p < 0.05; ***p < 0.001) according to two-way ANOVA followed by Tukey post-test to compare with the control (PB); no significant differences were found between the mutants and the WT form.

Skl and Pal have been described as DTT/β-mercaptoethanol-dependent enzymes (Sheehan et al., 1997; Llull et al., 2006), an effect that could denote reversal of catalytic thiol oxidation by the reducing agent. To test disulfide formation and, if produced, identify the relevant cysteine residues, non-catalytic cysteines of Pal and Skl (Figure 1A) were substituted by serine and preservation of native fold in purified mutants was assessed (Supplementary Figure 8). The SDS-PAGE mobilities of WT forms and mutants were compared under reducing (SB_β_ buffer) and non-reducing (SB buffer) conditions after blocking free thiols present in native samples with iodoacetamide (see section “Materials and Methods”). In SB_β_ buffer all variants migrated as a single band each (band A) at the position expected for the respective monomers (Figures 8A, 9A). However, in SB buffer Pal C250S, Skl C36S, and the WT endolysins gave also an additional band with slightly higher mobility (band B), which was indicative of a monomer with an intramolecular disulfide bridge. The absence of band B in lanes of Pal C34A and C112S and of Skl C125S pointed to monomers containing Cys34–Cys112 (Pal) or Cys31–Cys125 (Skl) intramolecular disulfide bridges. Additionally, a band with the mobility expected for a dimer was found in lanes of Pal C112S and C112S–C250S mutants in SB (Figures 8A–C), unveiling the ability of catalytic Cys34 to form a Cys34–Cys34 inter-molecular disulfide bridge when Cys112 is changed to serine (Figure 8A). A faint band corresponding to an Skl dimer with a Cys31–Cys31 intermolecular disulfide appeared also in the lane of Skl C36S–C125S in SB (Figures 9A–C). Thus, reduction of disulfide bridges might contribute to Pal and Skl activation by DTT or β-mercaptoethanol.

#### Bacteriolytic Action and Enhancement by DTT

Next, the activity with and without DTT of all cysteine variants was compared using the pneumococcal R6 strain as substrate. Addition of 10 mM DTT (PB_*DTT10*_) slightly increased the velocity of lysis by the WT forms (Figures 8B, 9B), in accordance with the activation seen on purified R6 cell walls (Supplementary Table 3). Unexpectedly, the lytic activity of Pal mutants C112S, C250S, and C112S–C250S in PB was much lower than that of Pal WT and the drop was largely reversed in PB_*DTT10*_ (Figures 8B,C). Addition of 10 mM DTT also unveiled the sigmoidal character of their time-course profiles, with lag duration augmenting in the order C112S-C250S < C112S < C250S. Moreover, increasing the time of treatment showed that the low activity displayed by these mutants in PB during the first 60 min of incubation corresponded to the initial phase of the respective sigmoid, as illustrated in Figure 8D for C250S mutant. Actually, OD_550_ rapidly decreased after the lag phase, approaching the final values attained in PB_*DTT10*_ (differences in Figure 8D final values arose from correction for control decay; see Supplementary Figure 7E). Accordingly, bacterial killing after 60-min treatment in PB_*DTT10*_ was similar for all Pal variants (2–3 logs of viable bacteria; Figure 8C), and the same happened in PB after 3-h exposure (2.5–3.5 logs of viable bacteria; Figure 8E). Interestingly, the shape of the time-course profile in PB_*DTT10*_ was the same with and without preincubation of the Pal mutant in this buffer (Figure 8B), evidencing that reduction of reversibly oxidized forms should be a fast process. However, the lag period of samples preincubated in PB_*DTT10*_ sharply depends on the concentration of DTT in the reaction media (Figure 8D) and the maximum velocity of lysis increased linearly with it up to 1 mM DTT (data not shown). Nonetheless, reduction of bacterium viability after 3-h treatment with Pal C250S was the same with and without DTT, being comparable or even higher than after treatment with Pal WT under identical experimental conditions.

In Skl, replacement of Cys36 and/or Cys125 with serine moderately decreased the rate of R6 lysis (C36S > C125S > C36S–C125S) and slightly increased the lag duration (Figure 9B), making evident the initial phase of the sigmoid in the profile of C36S–C125S mutant in PB. Stimulation of maximum activity by DTT was also moderate (∼2-fold in C125S and C36S–C125S mutants; ∼1.3 in Skl WT; almost null in Skl C36S). The steepest decrease in OD_550_ was attained in less than 10 min in both buffers for all variants and, consequently, slight-to-moderate variations in lethality were found after 60-min treatment (2.8–3.5 logs in PB vs. 3.5–4.3 logs in PB_*DTT*_; Figure 9C).

In summary, our results evidenced the sigmoidal character of Pal- and Skl-mediated bacterial lysis; the latency period increasing as the lytic activity decreases because of variations in enzyme concentration, reaction conditions, or sequence mutations in the catalytic domain or the CBD. Analogous effects have been described for the sigmoidal profiles of other endolysins with catalytic domains of different types and varying mechanisms of hydrolysis (Mayer et al., 2011; McGowan et al., 2012; Schmelcher et al., 2012; Dunne et al., 2016).

The PG layer is a dynamic structure with considerable plasticity and endolysins need to penetrate its intricate network to cleave the substrate. Target bonds may be relatively easy to access in some cell walls, while in others the enzyme has to expose the scissile bonds as the hydrolysis proceeds, likely sampling a substrate ready conformation. In this context, it seems plausible to hypothesize that a slow cell wall penetration might underlie the sigmoidal character of the lysis profiles. The lag phase would represent the random hydrolysis of easily accessible target bonds, with a reduced impact on OD_550_ values if the number of cleaved bonds was below the minimum required to achieve bacterial rupture (Mitchell et al., 2010). Loosening of PG packing upon the initial cleavage of stem peptides would increase the accessible concentration of scissile bonds and/or the fraction of productive binding for catalysis. Accordingly, the maximum rate of hydrolysis—corresponding to the pseudo-linear phase of the sigmoid—would be reached after a certain time of enzyme addition. Indeed, the strong activation seen after the lag phase in the profiles of Pal less active mutants may denote a better penetration of the PG layer as bridging of the polysaccharide chains is reduced by initial cleavage of stem peptides, thereby facilitating the formation of catalytically competent enzyme-substrate complexes. In Pal and Skl, this would likely require productive binding of the two catalytic modules housed by the dimers. On the other hand, the increase of the reaction rate would reduce the time required to form holes above the critical threshold for membrane prolapse and eventual cell rupture (Mitchell et al., 2013). In this way, the faster the initial cleavage of target bonds proceeds, the faster endolysin-mediated lysis will attain maximal activity, accounting for the factors found to affect the lag duration in Pal- and Skl-mediated lysis. Under optimal reaction conditions, the initial exponential phase can be too short to be detectable, as it likely happens, for example, in the lysis of R6 at 5 μg/ml of Pal or Skl WT in PB and PB_*DTT10*_ at pH 6.8; or with D39 strain at 1 μg/ml Skl WT in PB_*DTT10*_ at pH 6.0.

Consistently with our hypothesis, the velocity of lysis of *Streptococcus agalactiae* strain 333 cells by the B30 endolysin depends upon the growth stage at which bacteria were harvested. The increase of lysis resistance is associated with the appearance of an increasingly long period of latency (Pritchard et al., 2004), likely due to the changes experienced by the cell wall along the cell cycle. Also, the CHAP domain of the three-domain PlyTw endolysin has been proposed to dig and expose the PG bonds susceptible to the action of its *Amidase-2* domain, in order to explain the digestion products formed in the lysis from without with the full-length protein, because constructs lacking the CHAP domain showed no lytic activity (Becker et al., 2014). The possibility that endolysin dynamics could play also an important role in controlling bacteriolysis by Skl and Pal cannot be discarded at the moment. However, a conformational change of the enzyme, either free or attached to the PG, could not by itself explain current results because in such event the latency period duration would not depend on endolysin concentration.

Finally, our results evidenced that other factors than reversal of cysteine oxidation underlie the enhancement of Pal and Skl activities by DTT. The observed effect might reflect changes promoted by DTT in the pneumococcal envelope facilitating bacterial lysis. Addition of the reducing agent will change the external oxidoreduction potential, which, in turn, may modify the thiol-disulfide balance of proteins in the bacterial surface and the membrane permeability, with very different effects depending on the specific properties of the cell wall and membrane, among other factors, even in resting cells (Riondet et al., 1999; Waché et al., 2002). On the other hand, DTT might participate as well in the hydrolysis reaction acting as a nucleophile in deacylation of the thioester intermediate (Figure 1B), as both DTT and β-mercaptoethanol have been shown to convert peptide-^α^thioesters in peptide-^α^(DTT/β-mercaptoethanol) derivatives (Gates et al., 2013).

## Conclusion

Several key insights arise from this work. First, we have shown, for the first time, that Amidase_5 domains are cysteine-peptidases whose catalytic triad and fold supported their inclusion within Clan CA of cysteine-peptidases with a papain-like fold. Second, 3D *in silico* models of Pal and Skl endolysins have been generated, the substrate-binding grooves inferred, and residues relevant for substrate recognition and/or intermediate stabilization proposed. Additionally, six sequence/structurally conserved choline-binding loci have been identified from sequence analysis and homology modeling of CBDs, and the dimerization mode promoted by choline binding characterized. Third, our results showed a similar fold of Pal and Skl catalytic domains, and of CBDs, as well as that the overall 3D architecture of choline-bound dimers is preserved in all the choline-dependent pneumococcal lysins characterized to date−regardless of their catalytic domains and bond specificities−which suggests an adaptation to the host-specific PG structure. Fourth, we have shown that Skl is a good antibacterial agent against *S. pneumoniae* strains, its lethality comparing well with those of Pal and LytA. Besides, our results help to understand how Pal and Skl may accede to the target bonds in the lysis from without of *S. pneumoniae*, and revealed also that activation of both cysteine-peptidases by DTT does not take place, solely, through reversal of catalytic-cysteine oxidation. Understanding these processes at molecular level is key in our comprehension of how endolysin efficiency as antibacterials is finely tuned by the overall cell-wall structure of a given pathogen, of relevance in the clinical or industrial settings.

## Data Availability Statement

The datasets presented in this study can be found in online repositories. The names of the repository/repositories and accession number(s) can be found in the article/Supplementary Material.

## Author Contributions

MM and PG: conceptualization and funding acquisition. CG-P, RMB, NH-O, GG, PR-L, JFD, and PG: investigation. CG-P, RMB, NH-O, and JFD: formal analysis and visualization. MM: supervision and writing of the original draft. CG-P, RMB, NH-O, PG, and MM: manuscript review and editing. All authors have read and agreed to the final version of the manuscript.

## Conflict of Interest

The authors declare that the research was conducted in the absence of any commercial or financial relationships that could be construed as a potential conflict of interest.

## Publisher’s Note

All claims expressed in this article are solely those of the authors and do not necessarily represent those of their affiliated organizations, or those of the publisher, the editors and the reviewers. Any product that may be evaluated in this article, or claim that may be made by its manufacturer, is not guaranteed or endorsed by the publisher.

## Footnotes

1
http://www.biotech.uconn.edu/auf/

2
https://predictprotein.org

3
bioinf.cs.ucl.ac.uk/psipred/

4
https://www.compbio.dundee.ac.uk/jpred4

5
http://zhanglab.ccmb.med.umich.edu

6
http://sysbio.rnet.missouri.edu/3Drefine

7
https://saves.mbi.ucla.edu

8
http://consurf.tau.ac.il

9
http://www.pymol.org
