# Supplementary material for: Structural and Functional Insights Into Skl and Pal Endolysins, Two Cysteine-Amidases With Anti-pneumococcal Activity. Dithiothreitol (DTT) Effect on Lytic Activity
Source: Front Microbiol. 2021 Oct 29;12:740914. doi: 10.3389/fmicb.2021.740914 (PMC8586454; doi:10.3389/fmicb.2021.740914)
Supplement: Supplementary file 1 [file Presentation_1.pdf]

Supplementary Material

Structural and Functional Insights into Skl and Pal Endolysins, Two Cysteine-amidases with Anti-pneumococcal Activity. Dithiothreitol (DTT) Effect on Lytic Activity

Cristina Gallego-Páramo[1,2], Noelia Hernández-Ortiz[1], Rubén M. Buey[3], Palma Rico-Lastres[1,2], Guadalupe García[1,2], J. Fernando Díaz[4], Pedro García[2,4], Margarita Menéndez[1,2*]

[1]Instituto de Química-Física Rocasolano, Consejo Superior de Investigaciones Científicas, Madrid, Spain.
[2] Centro de Investigación Biomédica en Red de Enfermedades Respiratorias (CIBERES), ISCIII, Madrid, Spain.
[3] Metabolic Engineering Group, Universidad de Salamanca, Salamanca, Spain.
[4]Centro de Investigaciones Biológicas Margarita Salas, Consejo Superior de Investigaciones Científicas, Madrid, Spain.

Content:

Supplementary Figures: 8

Supplementary Tables: 3

Supplementary References

The data sets presented in this study, including supplementary material can be found in the FigShare repository (https://doi.org/10.6084/m9.figshare.c.5613674). Further information can be requested to the authors.

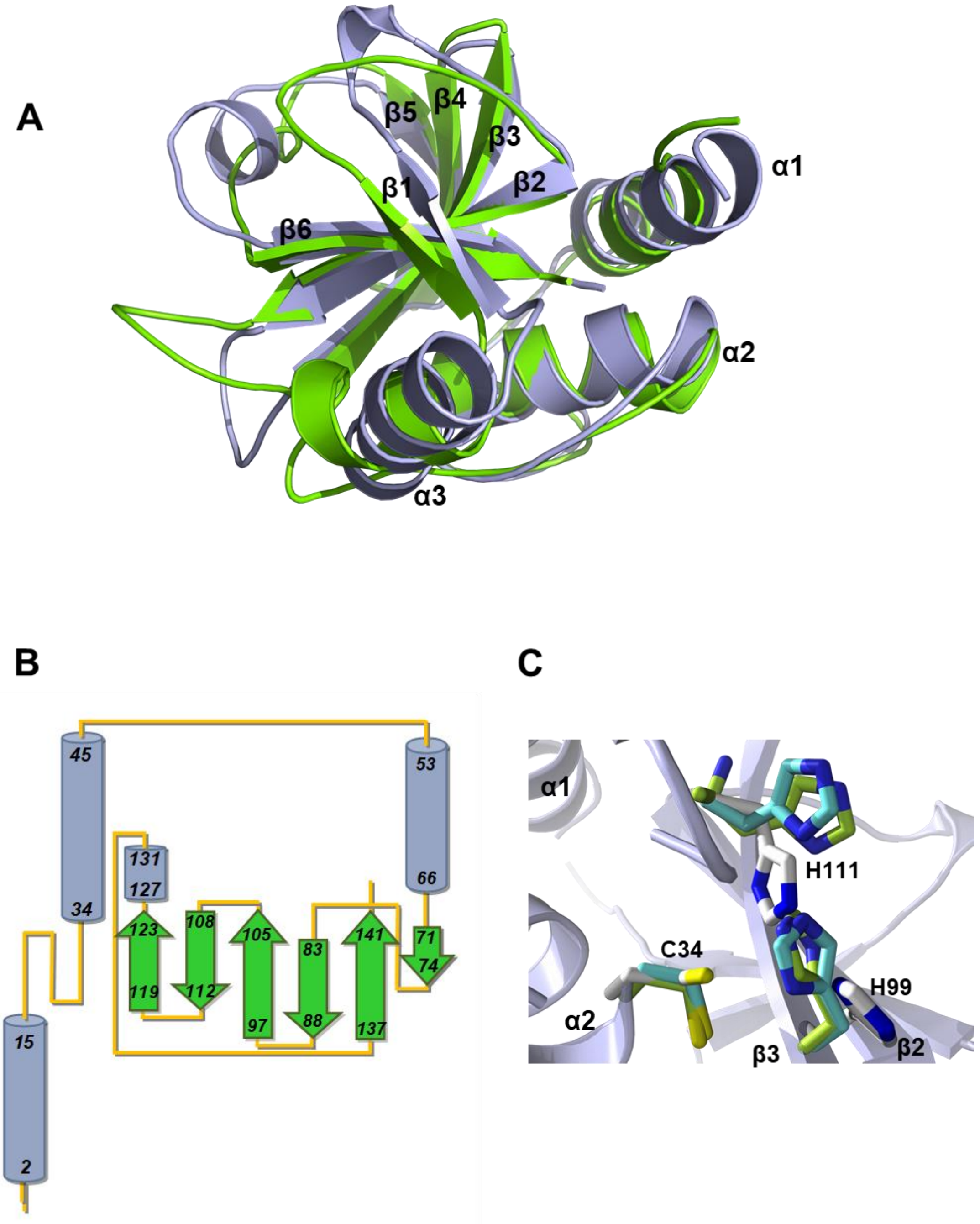

Supplementary Figure 1. Structural model of N-Pal generated by ITASSER. (A) Structural superimposition of models built with I-TASSER and MODELLER (cartoon representation) colored in light blue and green, respectively. **(B)** Topology diagram of the I-TASSER model. **(C)** Close-up view of Pal catalytic residues in the ITASSER model (stick representation; C: white, N: blue, S: yellow) *vs* disposition in the MODELLER model (C: green, N: blue, S: yellow) and the 2EVR structure (C: cyan, N: blue, S: yellow).

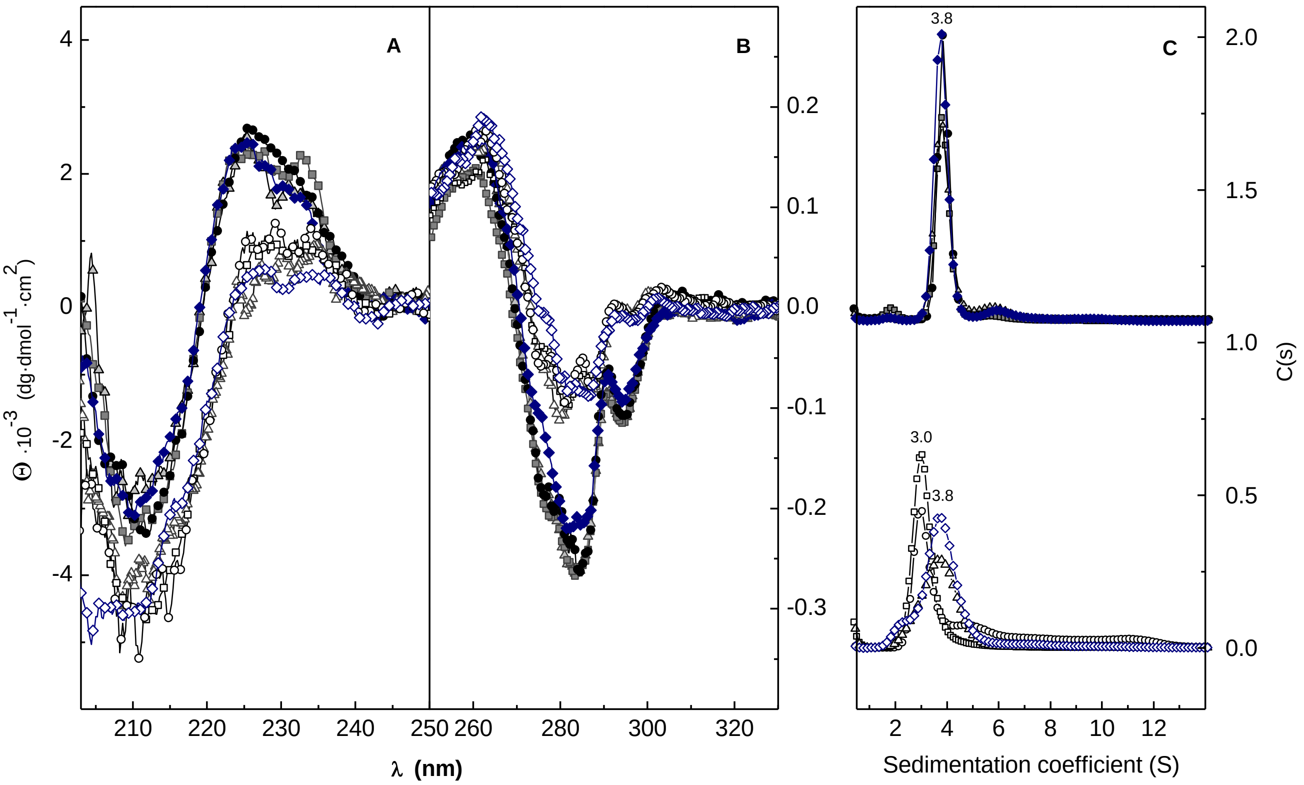

Supplementary Figure 2. Structure conservation in Pal catalytic mutants. **(A, B)** Far- and near-UV CD spectra in the absence and presence of 80 mM choline (open and full symbols, respectively). **(C)** Distribution of sedimentation coefficients with (top traces) and without (bottom traces) 80 mM choline. Pal WT: circles; C34A: squares; H99A: triangles; and H111A: blue diamonds. Data acquired in PB buffer, pH 8.0, at 20 °C. The C34A mutant is a full monomer without choline and a dimer in its complexed form. In the unbound form of Pal WT and the other two mutants several species coexist in variable proportion, but choline binding leads in all cases to dimer formation (figure labels indicate $s_{20,w}$ values).

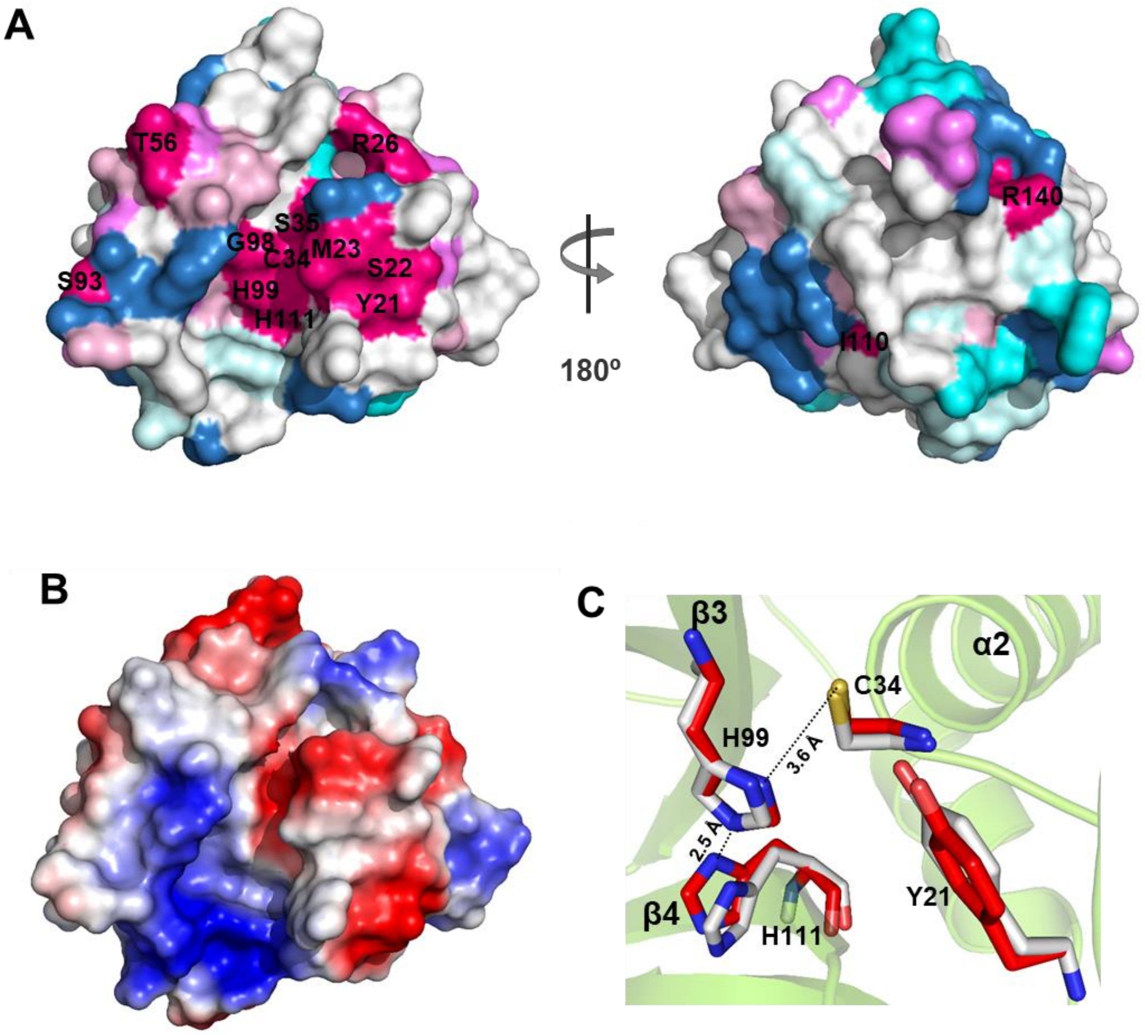

Supplementary Figure 3. N-Pal surface and catalytic site overview. **(A)** Surface representation of the substrate-binding cleft (left) and the opposite face (right) with sequence conservation scores from ConSurf mapped onto the surface; from deep magenta (highly conserved) to dark blue (highly variable). **(B)** Electrostatic-potential surface generated by PyMOL APBS tool for the active-site side of N-Pal colored from negative (red) to positive (blue)**. (C)** Comparative disposition of catalytic residues (stick representation; Pal sequence numbering) in the MODELLER model (C: red, N: blue, S: yellow) and the 2EVR template (C: white, N: blue, S: yellow). Feasible polar interactions are depicted as dotted lines. The distance between the sulphur atom of Cys34 and the Nδ1 atom of His99 compares well with the distance found in 2EVR and NlpC/P60 domains accessible in the PDB (average value, 3.7 ± 0.2 Å).

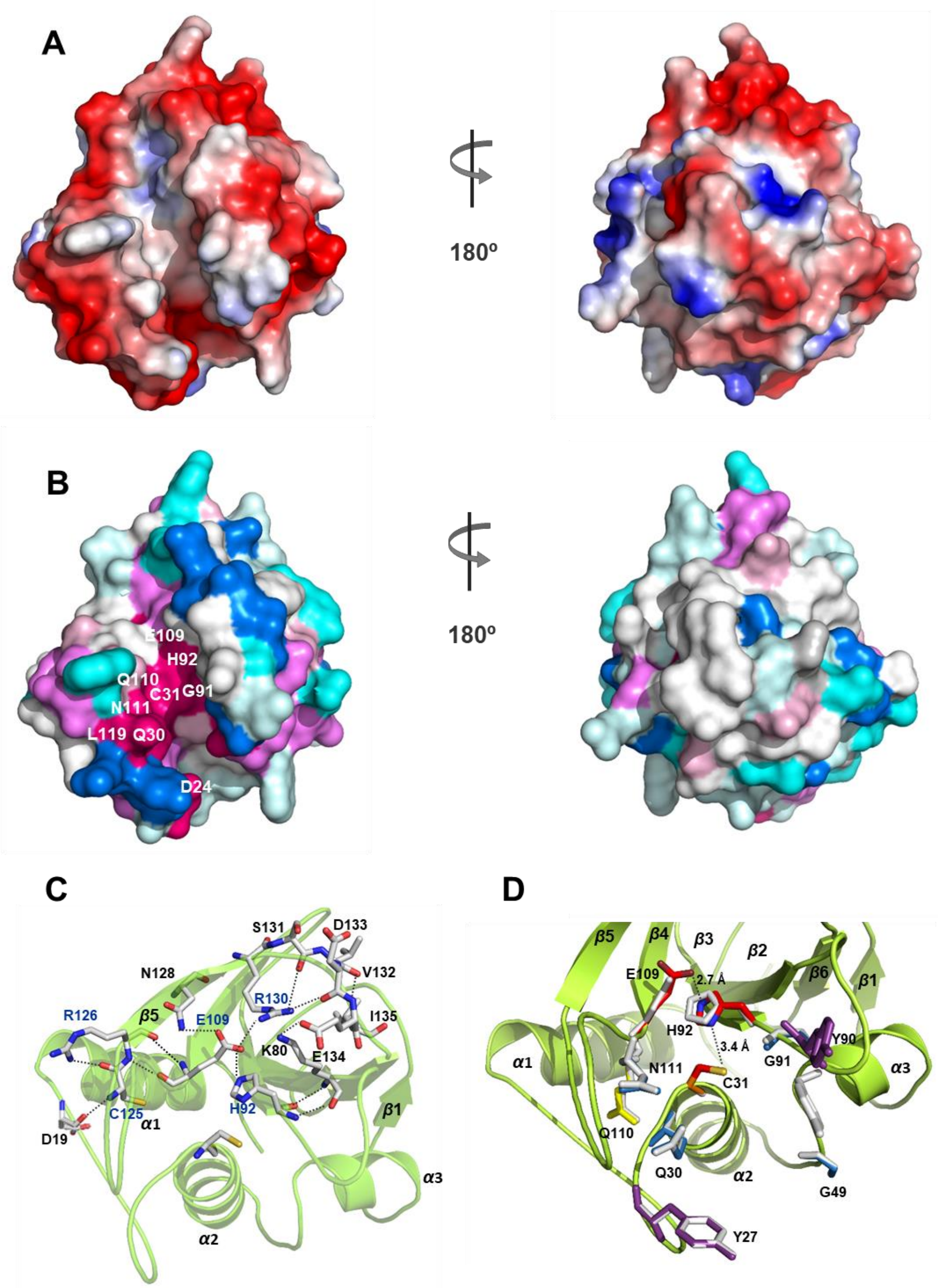

Supplementary Figure 4. N-Skl surface and catalytic site. (A) Electrostatic potential surface generated by PyMOL for the substrate-binding face (left) and the opposite side (right) colored from negative (red) to positive (blue). **(B)** Sequence conservation on N-Skl surface depicted by residue coloring according to Consurf code (orientation as in (A)). **(C)** Network of feasible hydrogen bonds centered by Glu109 side chain. **(D)** Detailed view of Skl active cavity superimposed with the 6IST template. Residues relevant for activity are depicted in stick representation (Skl numbering): catalytic residues in red; residues likely involved in *i*) His92 charge distribution in yellow, *ii*) substrate binding and anionic intermediate stabilization in blue; and *iii*) substrate entrance in purple. Equivalent residues in 6IST template are in grey.

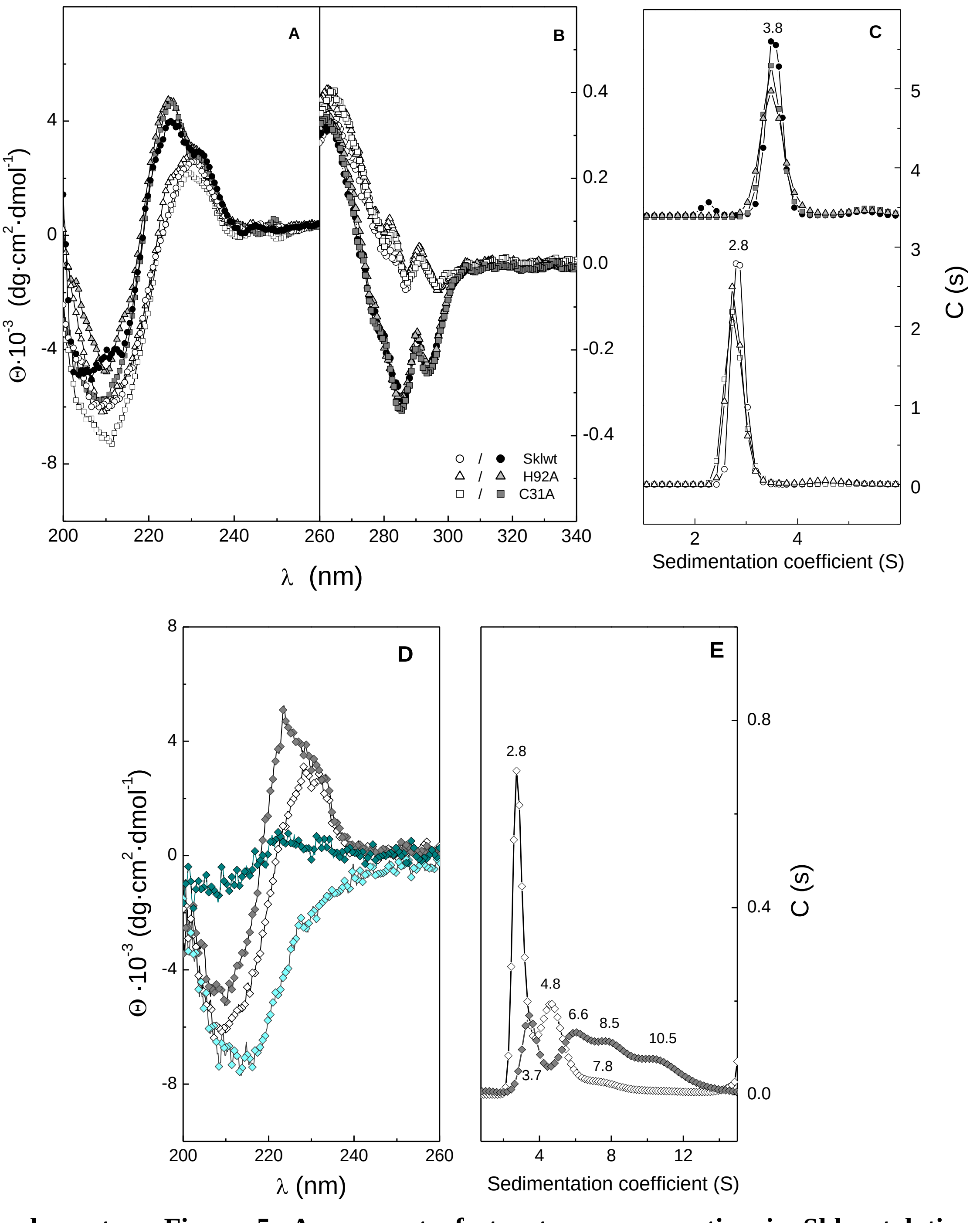

Supplementary Figure 5. Assessment of structure conservation in Skl catalytic mutants. (A, B) Far- and near-UV CD spectra of Skl WT (circles) and C31A (squares) and H92A (triangles) mutants in the absence and presence of 300 mM choline (open and full symbols, respectively). **(C)** Sedimentation coefficients distribution of Skl WT, C31A and H92A with (top traces) and without (bottom traces) 300 mM choline. Symbols as in (A). **(D)** Far-UV CD spectra of recently dialyzed E109A mutant in the absence and presence of 300 mM choline (white and grey symbols, respectively) and after several hours at 20 °C (light and dark cyan, respectively). **(E)** Distribution of sedimentation coefficients of E109A mutant with and without 300 mM choline (grey and white diamonds, respectively. (C) and (E) figure labels indicate $s_{20,w}$ values. Measurements made in PB, pH 6.5, at 20 °C.

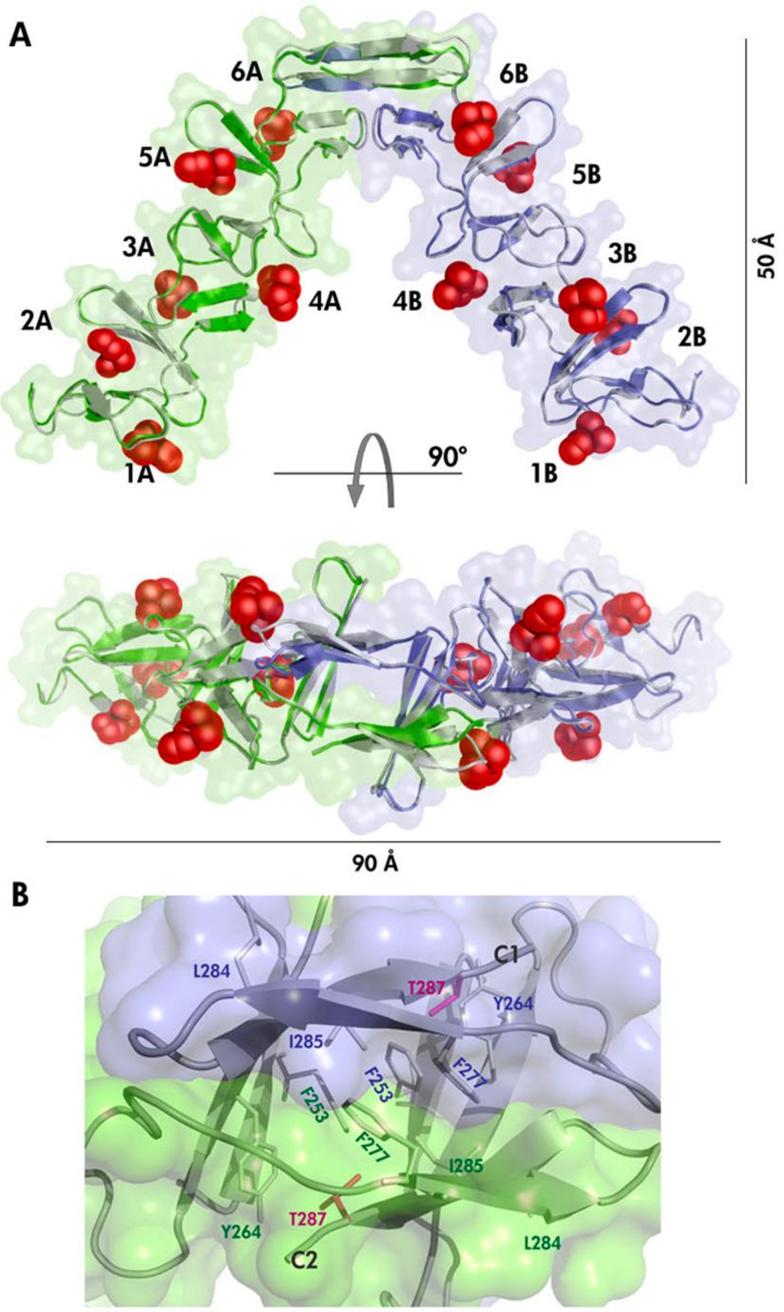

Supplementary Figure 6. Dimerization mode of Skl. (A) Disposition of two C-Skl monomers (in green and blue) in the model of the C-Skl dimer built by structural superimposition with the C-LytA dimer (PDB access code: 4IWT) that was used in rigid-body modeling of the SAXS-based overall structure of Skl dimer. Choline moieties, in red-sphere representation, are numbered according to their position in each monomer. **(B)** Dimerization interface with amino acids involved in the hydrophobic core shown in stick representation. With exception of Thr287 (Val317 in LytA), they would be sequentially and structurally conserved in the Skl dimer. C1 and C2 indicate the C-terminus of the monomers.

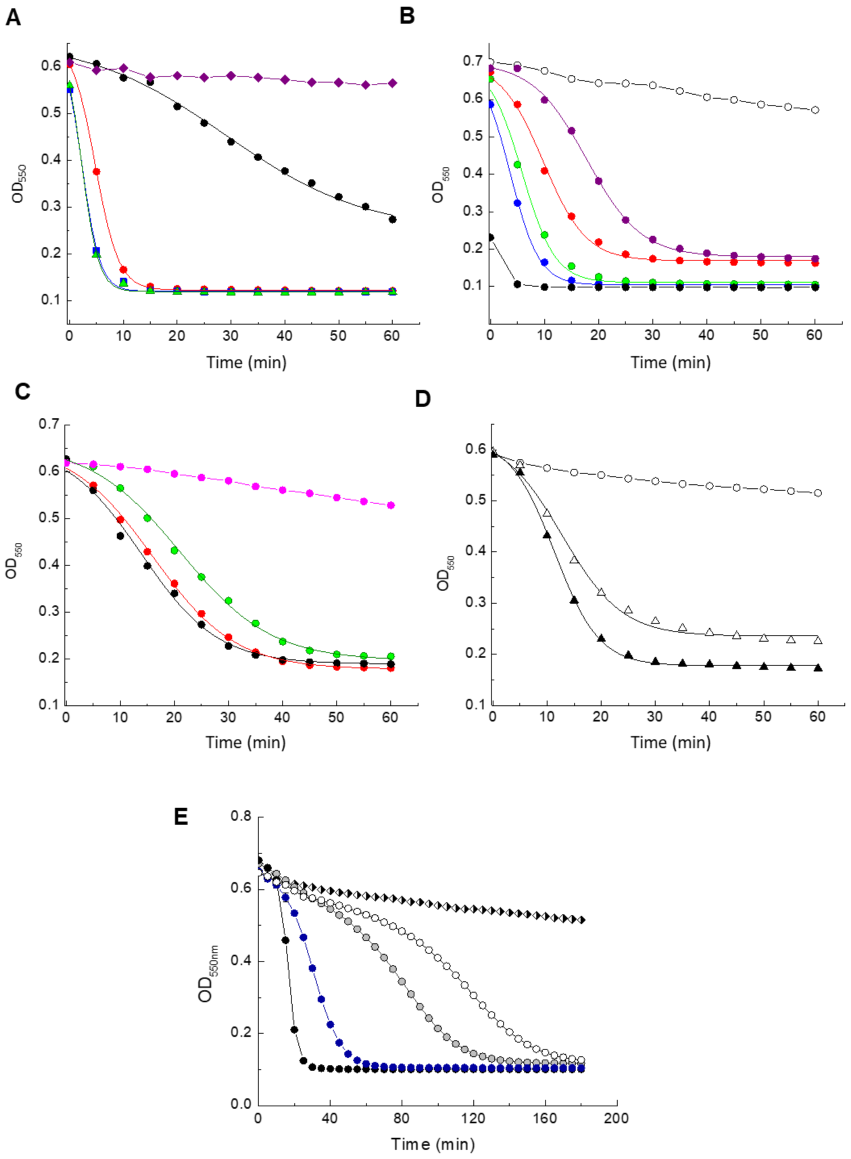

Supplementary Fig. 7. Sigmoidal character of *S. pneumoniae* bacteriolysis by Pal and Skl. (A) Dose-dependence of *S. pneumoniae* R6 lysis by Skl in PBS_{DTT10}, pH 6.8, 37 °C. Symbols represent experimental data after correction for control decay (0.1 µg/ml: purple; 1 µg/ml: black; 5 µg/ml: red; 15 µg/ml: blue; 30 µg/ml green), and solid lines at 1–30 µg/ml the fit to the Boltzman sigmoid. **(B)** Effect of NaCl concentration on the lysis profiles of strain D39 by Skl (1 µg/ml; PB_{DTT10}, pH 6.0, 37 ºC) after correction for control decays (NaCl concentrations: 0 mM (black); 50 mM (blue); 100 mM (green); 150 mM (red); 200 mM (magenta). **(C)** pH influence on D39 lysis by Skl (1µg/ml) at 37 °C in Ac_{DTT10} buffer at pH 5.5 (magenta) and pH 6.0 (green), and in PB_{DTT10} at pH 6.5 (red) and pH 6.0 (black). **(D)** Lysis profiles of the R6 strain by Pal H111A (5 µg/ml) in PB (open triangles) and PB_{DTT10} (solid triangles) at 37 °C. Symbols and solid lines in B, C and D mean as in (A); open black circles in (B) and (D) illustrate control decay. **(E)** Experimental profiles of R6 lysis by Pal C250S mutant displayed in Fig. 8D without correction for control decay. Control: white-black diamonds; no-DTT (-): white circles; 0.1 mM DTT: grey circles; 1 mM DTT: blue circles; 10 mM DTT: black symbols. Panels depict representative results.

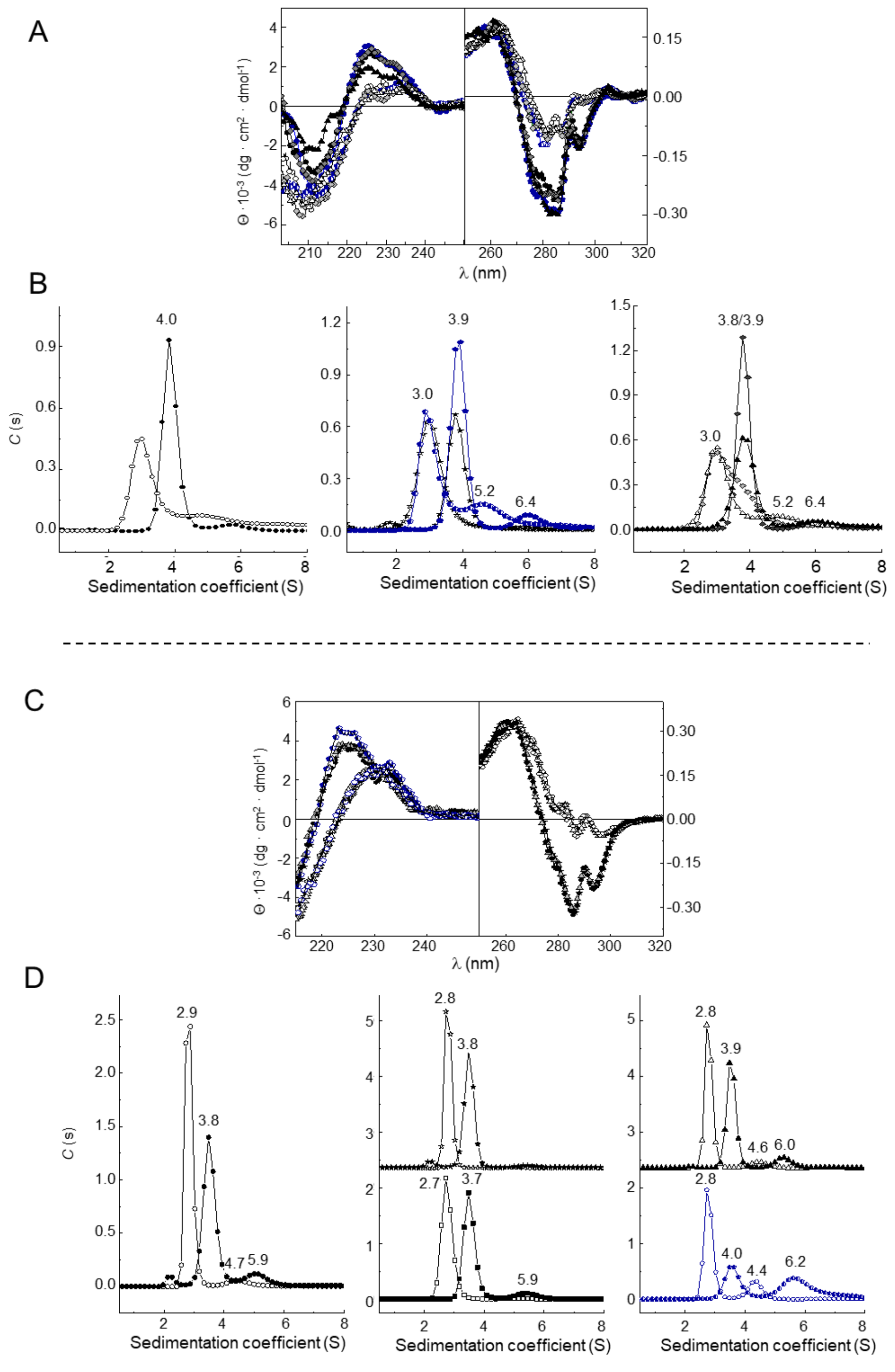

Supplementary Figure 8. **Pal and Skl structural conservation in Cys→Ser mutants. (A)** Far- and near-UV CD spectra and **(B)** sedimentation coefficient distribution of Pal WT (circles), C34A (stars), C112S (pentagons), C250S (diamonds) and C112S/C250S (triangles) mutants in the absence (open symbols) and presence (solid symbols) of 80 mM choline (PB, pH 8.0, 20 °C). **(C)** Far- and near-UV CD spectra and **(D)** sedimentation coefficient distribution of Skl WT (circles), C31A (squares), C36S (stars), C125S (triangles) and C36S–C125S (pentagons) mutants in the absence and presence (open and solid symbols, respectively) of 300 mM choline (PB, pH 6.5, 20 °C). (B) and (D) figure labels indicate $s_{20,w}$ values.

Supplementary Table 1. Strains used in this study

**Organism**	Strain	Notes	Reference
Streptococcus pneumoniae	R6	Laboratory D39 derivative; Non-encapsulated	Hoskins et al., 2001
	D39	Serotype 2	Lanie et al., 2007
	48	Multidrug resistant strain. Serotype 23F	Ramos-Sevillano et al., 2012
	2963/13	Non-PCV13[a]. Serotype 11A	Domenech et al., 2015
	2896/13	Non-PCV13. Serotype 35B	Domenech et al., 2015
Escherichia coli	DH5α	Bacterium host for cloning	
	DH10B	Bacterium host for cloning	Durfee et al., 2008
	BL21(DE3)	Bacterium host for protein overproduction	Studier and Moffat, 1986.
	C41(DE3)	Bacterium host for protein overproduction	Dumon-Seignovert et al., 2004

[a]Non-PCV13 stands for serotype non included in the 13-valent pneumococcal conjugate vaccine.

Supplementary Table 2. Oligonucleotides and plasmids used in this study

Oligonucleotides	Sequence[a]
Pal5 Fw	5’-GGTCTAGAGGTGGAATAATGGG-3’
DL2 Rv	5’-GGAAGCTTAAACTTTAGCAGTAATGAG-3’
C34A Fw	5’- CGGTCCTGATAGCTATGAC**GCA**TCAAGTTCTATGTACTATGC-3’
C34A Rv	5’- GCATAGTACATAGAACTTGA**TGC**GTCATAGCTATCAGGACCG-3’
H99A Fw	5’-GCAGGCGCTGGAGGT**GCA**ACAGGGATGTTCATTGACAG-3’
H99A Rv	5’-CTGTCAATGAACATCCCTGT**TGC**ACCTCCAGCGCCTGC-3’
H111A Fw	5’-GACAGTGATAACATCATT**GCC**TGCAACTACGCCTACGACG -3’
H111A Rv	5’-CGTCGTAGGCGTAGTTGCA**GGC**AATGATGTTATCACTGTC-3’
C112S Fw	5’-CAGTGATAACATCATTCAC**AGC**AACTACGCCTACGACGG-3’
C112S Rw	5’-CCGTCGTAGGCGTAGTT**GCT**GTGAATGATGTTATCACTG-3’
C250S Fw	5’-CGATAATTGGTATTAT**TCT**GATGCTACCAACGGCGAC-3’
C250S Rw	5’-GTCGCCGTTGGTAGCATC**AGA**ATAATACCAATTATCG-3’
C36S Fw	5’-GTGCGCTGACGTACCT**AGT**TACGGTCTTCGTC-3’
C36S Rw	5’-GACGAAGACCGTA**ACT**AGGTACGTCAGCGCAC-3’
C125S Fw	5’-GAAGTAGGTGGCCCT**AGT**CGCTACAATGAGCG-3’
C125S Rw	5’-CGCTCATTGTAGCG**ACT**AGGGCCACCTACTTC-3’

Plasmids	Description	Overexpression (Strain/Temperature/[IPTG])
pMSP11[b]	pIN-III-A3 derivative containing the *Pal* gene, Amp^R	DH5α/37 °C/400 µM
pPal	pT7-7 derivative containing the *Pal* gene	
pPal-C34A	pT7-7 derivative encoding Pal C34A mutant	BL21(DE3)/24 °C/500 µM
pPal-H99A	pT7-7 derivative encoding Pal H99A mutant	BL21(DE3)/24 °C/500 µM
pPal-H111A	pT7-7 derivative encoding Pal H111A mutant	C41(DE3)/24 °C/400 µM
pPal-C112S	pT7-7 derivative encoding Pal C112S mutant	C41(DE3)/37 °C/400 µM
pPal-C250S	pT7-7 derivative encoding Pal C250S mutant	C41(DE3)/37 °C/400 µM
pPal-C250S–C112S	pT7-7 derivative encoding Pal C112S-C250S double mutant	C41(DE3)/37 °C/400 µM
pFSK6[c]	pIN-III-A3 derivative containing the *Skl* gene	BL21(DE3)/30 °C/200 µM
pSkl	pT7-7 derivative containing the *Skl* gene	BL21(DE3)/30 °C/200 µM
pSkl-C31A	pT7-7 derivative encoding Skl C31A mutant	BL21(DE3)/30 °C/100 µM
pSkl-H92A	pT7-7 derivative encoding Skl H92A mutant	BL21(DE3)/30 °C/100 µM
pSkl-E109A	pT7-7 derivative encoding Skl E109A mutant	BL21(DE3)/30 °C/100 µM
pSkl-C36S	pT7-7 derivative encoding Skl C36S mutant	BL21(DE3)/30 °C/100 µM
pSkl-C125S	pT7-7 derivative encoding Skl C125S mutant	BL21(DE3)/30 °C/100 µM
pSkl-C36S–C125S	*p*T7-7 derivative encoding Skl C36S-C125S double mutant	BL21(DE3)/30 °C/100 µM
pFSK-Y264H	pIN-III-A3 derivative encoding Skl Y264H spontaneous mutant, Amp^R	BL21(DE3)/30 °C/50 µM
pT7-7[d]	Expression vector, Amp^R	

[a] Underlining indicates restriction sites for cloning purposes; in bold, codon modified for amino acid mutation. [b]Sheehan. [b]Sheehan et al., 1997. [c]Llull et al., 2006. [d]Tabor and Richarson, 1995.

Supplementary Table 3. Relative activity of Pal and Skl variants on pneumococcal cell walls[a]

Skl			Pal		
variant	%		variant	%	
	(-)	(+)		(-)	(+)
WT	71 ± 20	100	WT	80 ± 16	100
C31A	ND	6 ± 1	C34A	0.008±0.006	0.008 ± 0.007
H92A	ND	1.4 ± 0.5	H99A	2.8 ± 0.5	3.8 ± 0.7
			H111A	15 ± 4	36 ± 5
C36S	51 ± 5	59 ± 3	C112S	63 ± 6	99 ± 20
C125S	54 ± 20	94 ± 7	C250S	19 ± 3	36 ± 6
C36S-C125S	23 ± 2	55 ± 2	C112S-C250S	25 ± 3	26 ± 2

[a] Measurements made in PB (–) or PB_{DTT10} (+), pH 6.8, at 37 °C

SUPPLEMENTARY REFERENCES

Domenech, M., Damián, D., Ardanuy, C., Liñares, J., Fenoll, A., and García, E. (2015). Emerging, non-PCV13 serotypes 11A and 35B of *Streptococcus pneumoniae* show high potential for biofilm formation *in vitro*. PLoS One. 10(4): e0125636.

Dumon-Seignovert, L., Cariot, G., and Vuillard, L. (2004). The toxicity of recombinant proteins in *Escherichia coli:* a comparison of overexpression in BL21(DE3), C41(DE3), and C43(DE3). Protein expression and purification 37, 203–206.

Durfee, T., Nelson, R., Baldwin, S., Plunkett III, G., Burland, V., Mau, B., et al. (2008). The complete genome sequence of *Escherichia coli* DH10B: insights into the biology of a laboratory workhorse. J. Bacteriol. 190, 2597–2606.

Hoskins, J., Alborn, W.E., Arnold, J., Blaszczak, L.C., Burgett, S., DeHoff, B.S., et al. (2001). Genome of the bacterium *Streptococcus pneumoniae* strain R6. J. Bacteriol. 183, 5709–5717.

Lanie, J.A., Fu, D.-J., Kamierczak, K.M., Andrezejewski, T.M., Davidsen, T.M., Wayne, K.J., et al. (2007). Genome sequence of Avery's virulent serotype 2 strain D39 of *Streptococcus pneumonia*e and comparison with that of unencapsulated laboratory strain R6. J. Bacteriol. 189, 38–51.

Llull, D., López, R., and García, E. (2006). Skl, a novel choline-binding *N*-acetylmuramoyl-L-alanine amidase of *Streptococcus mitis* SK137 prophage containing a CHAP domain. FEBS Lett. 580, 1959–1964.

Ramos-Sevillano, E., Rodríguez-Sosa, C., Díez-Martínez, R., Olmedillas, M.-J., García, P., García, E., et al. (2012). Macrolides and β-lactam antibiotics enhance c3b deposition on the surface of multidrug-resistant *Streptococcus pneumonia*e strains by a LytA autolysin-dependent mechanism. Antimicrob. Agents Chemother. 56, 5534–5540.

Sheehan, M.M., García, J.L., López, R., and García, P. (1997). The lytic enzyme of the pneumococcal phage Dp-1: a chimeric lysin of intergeneric origin. Mol. Microbiol. 25, 717–725.

Studier, F.W., and Moffat, B.A. (1986). Use of bacteriophage T7 RNA polymerase to direct selective high-level expression of cloned genes. J. Mol. Biol. 189, 113–130.

Tabor, S., and Richardson, C.C. (1985). A bacteriophageT7 RNA polymerase/promoter system for controlled exclusive expression of specific genes. Proc. Natl. Acad. Sci. USA. 82, 1074–1078.
